# The Fecal Microbiome in Cats with Diarrhea

**DOI:** 10.1371/journal.pone.0127378

**Published:** 2015-05-19

**Authors:** Jan S. Suchodolski, Mary L. Foster, Muhammad U. Sohail, Christian Leutenegger, Erica V. Queen, Jörg M. Steiner, Stanley L. Marks

**Affiliations:** 1 Gastrointestinal Laboratory, Texas A&M University, College Station, TX, United States of America; 2 Department of Physiology, Government College University, Faisalabad, Pakistan; 3 IDEXX Reference Laboratories, West Sacramento, CA, United States of America; 4 MarQueen Animal Clinic, Roseville, CA, United States of America; 5 Department of Medicine & Epidemiology, School of Veterinary Medicine, University of California, Davis, CA, United States of America; University of Minnesota, UNITED STATES

## Abstract

Recent studies have revealed that microbes play an important role in the pathogenesis of gastrointestinal (GI) diseases in various animal species, but only limited data is available about the microbiome in cats with GI disease. The aim of this study was to evaluate the fecal microbiome in cats with diarrhea. Fecal samples were obtained from healthy cats (n = 21) and cats with acute (n = 19) or chronic diarrhea (n = 29) and analyzed by sequencing of 16S rRNA genes, and PICRUSt was used to predict the functional gene content of the microbiome. Linear discriminant analysis (LDA) effect size (LEfSe) revealed significant differences in bacterial groups between healthy cats and cats with diarrhea. The order Burkholderiales, the families Enterobacteriaceae, and the genera *Streptococcus* and *Collinsella* were significantly increased in diarrheic cats. In contrast the order Campylobacterales, the family Bacteroidaceae, and the genera *Megamonas*, *Helicobacter*, and *Roseburia* were significantly increased in healthy cats. Phylum Bacteroidetes was significantly decreased in cats with chronic diarrhea (>21 days duration), while the class Erysipelotrichi and the genus *Lactobacillus* were significantly decreased in cats with acute diarrhea. The observed changes in bacterial groups were accompanied by significant differences in functional gene contents: metabolism of fatty acids, biosynthesis of glycosphingolipids, metabolism of biotin, metabolism of tryptophan, and ascorbate and aldarate metabolism, were all significantly (p<0.001) altered in cats with diarrhea. In conclusion, significant differences in the fecal microbiomes between healthy cats and cats with diarrhea were identified. This dysbiosis was accompanied by changes in bacterial functional gene categories. Future studies are warranted to evaluate if these microbial changes correlate with changes in fecal concentrations of microbial metabolites in cats with diarrhea for the identification of potential diagnostic or therapeutic targets.

## Introduction

The use of next generation sequencing of 16S rRNA genes has vastly improved our understanding about the bacterial groups present in the gastrointestinal (GI) tract of various animal species including cats, dogs, and horses, and several studies have reported that these complex bacterial communities are altered in gastrointestinal inflammation [1–7]. The observed alterations in the GI microbiota bear similarities to the dysbiosis observed in humans or animal models with intestinal inflammation [8–10], suggesting that microbial dysbiosis in inflammatory conditions of the gut are conserved across mammals. The GI microbiota plays an important role for maintaining host health, by providing stimulatory signals to the immune system and gut structure, by providing defense mechanisms against transient enteropathogens, and by providing nutritional benefits to the host through production of various metabolites that can be utilized by the host (e.g., vitamins, volatile fatty acids). While recent studies have described changes in the gastrointestinal microbiota due to dietary modifications in cats [11], only a few studies have evaluated alterations in the intestinal bacterial communities in cats with GI disease, and most of these studies have focused on specific bacterial groups using fluorescence in-situ hybridization (FISH) [7,12–14]. In one study, cats with IBD had increased counts of *Desulfovibrio* spp. when compared to control cats [13]. In contrast, another study did not identify any significant differences in FISH bacterial counts between cats with IBD and controls, although similar bacterial groups were targeted [12]. A recent study utilized next generation sequencing and identified changes in fecal microbiota in cats with chronic diarrhea that correlated with therapeutic responses to dietary modifications, but no detailed comparisons to healthy cats were reported in that study [14]. Therefore, the aims of the current study were to profile the fecal microbiome using 16S rRNA sequencing and predict the functional potential of the microbiota using PICRUSt in healthy cats and cats with acute and chronic diarrhea [15].

## Materials and Methods

### Animal enrollment and sample collection

The samples analyzed in this study were obtained as part of a previously published study that evaluated the prevalence of selected bacterial and parasitic enteropathogens in feces from cats from Northern California [16]. A portion of the DNA from those samples was used in the current study. To summarize briefly, naturally passed fresh fecal samples were obtained from healthy control cats (n = 21) as well as cats with acute diarrhea (n = 14; AD, defined as duration of ≤ 21 days) or chronic diarrhea (n = 29; CD; defined as duration of > 21 days) and processed within 2–3 hours of collection. The healthy cats belonged to students and staff at the University of California, Davis, Veterinary Medical Teaching Hospital (VMTH). Samples from diseased cats were collected when the animals were presented either to local practitioners or board certified internists for work-up of GI disease or from diarrheic shelter cats. Cats with a history of recent anthelmintic or antibiotic administration were excluded from analysis. An aliquot of feces (3–5 g) was immediately refrigerated at 3°C after collection and processed for DNA extraction within 24 hours of collection at a reference laboratory (IDEXX Reference Laboratories, West Sacramento, CA). The study was reviewed by the UC Davis Clinical Trials Review Board (CTRB). Because only freely passed fecal samples were collected from patients and healthy cats, no specific ethical approval was required.

### DNA isolation

DNA was isolated from fecal samples as described in detail in previous publications [16,17]. Briefly, 1 g of fecal material was reconstituted in lysis solution (Xtractor Gene Liquid Sample Reagent Pack and Lysis Buffer, Sigma Aldrich) and incubated for 10 minutes. Lysates were centrifuged and supernatants were extracted using Whatman filters in a Corbett X-Tractor platform (Qiagen Inc).

### Sequencing of 16S rRNA genes

Sequencing of the V4 region of the 16S rRNA gene was performed at MR DNA (www.mrdnalab.com, Shallowater, TX, USA) on an Ion Torrent PGM following the manufacturer’s guidelines using forward and reverse primers: 515F (5’-GTGCCAGCMGCCGCGGTAA-3’) and 806R (5’- GGACTACVSGGGTATCTAAT-3’). Briefly, the PCR reaction was performed in a single-step 30 cycle PCR using the HotStarTaq Plus Master Mix Kit (Qiagen, USA) under the following conditions: 94°C for 3 minutes, followed by 28 cycles (5 cycles used on PCR products) of 94°C for 30 seconds, 53°C for 40 seconds and 72°C for 1 minute, after which a final elongation step at 72°C for 5 minutes was performed. After sequencing, barcodes and primers were removed from the sequences, then short (<150bp), ambiguous, homopolymeric, and chimeric sequences were depleted from the dataset. Operational Taxonomic Units (OTUs) were assigned based on at least 97% sequence similarity using the QIIME v1.7 pipeline [18]. Sequences were rarefied to an equal depth of 12,000 sequences per sample. The sequences were deposited in SRA under accession number SRP047088.

### Quantitative PCR

Quantitative PCR (qPCR) was used as described previously to evaluate total bacteria and specific bacterial groups that have been shown to be frequently altered in intestinal health (i.e., *Faecalibacterium* spp., *Escherichia coli*, and *Clostridium perfringens*) [1,19].

### Prediction of functional gene content

The software PICRUSt (Phylogenetic Investigation of Communities by Reconstruction of Unobserved States) was used to predict the functional gene content in the fecal microbiome based on the 16S rRNA genes found in the data and represented in the Greengenes phylogenetic tree of 16S rRNA gene sequences [15]. PICRUSt was used online in the Galaxy workflow framework.

### Statistical analysis

All datasets were tested for normality using the Shapiro-Wilk test (JMP 10, SAS software Inc.). Differences in bacterial communities between healthy cats and cats with diarrhea were analyzed using the phylogeny-based unweighted UniFrac distance metric and PCoA plots and rarefaction curves were generated within QIIME [18]. ANOSIM (Analysis of Similarity) within the software package PRIMER 6 (PRIMER-E Ltd., Luton, UK) was used to determine significant differences in microbial communities between healthy cats and diseased cats. Because most datasets did not meet the assumptions of normal distribution, statistical testing between healthy and disease cats were performed using non-parametric Kruskal-Wallis tests or a Mann-Whitney U test where applicable. The resulting p-values were adjusted for multiple comparisons using the Benjamini & Hochberg’s False Discovery Rate (FDR), and an adjusted p<0.05 was considered statistically significant [20]. A Dunn’s post-test was used to determine which disease types were significantly different. Linear discriminant analysis effect size (LEfSe) was used to elucidate bacterial taxa (16S rRNA genes) and functional genes (PICRUSt) associated with healthy or diseased cats. LEfSe was used online in the Galaxy workflow framework.

## Results

### Animal population

**Fig 1:**
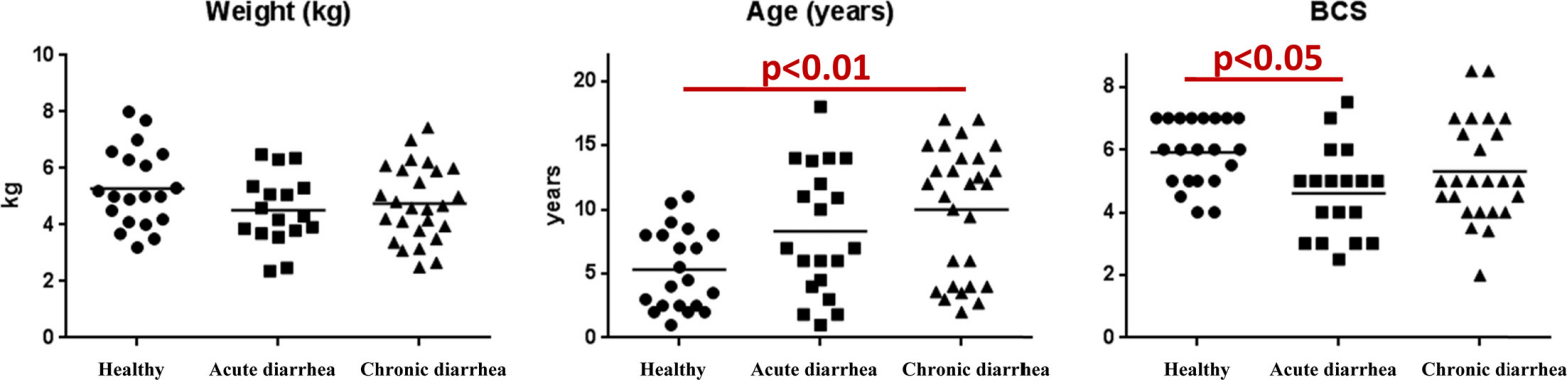
Body weight, age, and Body Condition Score (BCS) in evaluated cats.

No significant differences in body weights were identified between healthy cats (mean ± SD: 5.2 ± 1.4 kg) and cats with acute or chronic diarrhea (4.5 ± 1.2 or 4.8 ± 1.3, respectively; Fig 1). A significant difference (p<0.01) in age was observed between healthy cats (5.3 ± 3.2 years) and cats with chronic diarrhea (10.0 ± 5.0 years). Furthermore, healthy cats had a significantly (p<0.05) higher Body Condition Scores (BCS) compared to cats with acute diarrhea (5.9 ± 1.1 vs 4.6 ± 1.4; Fig 1).

### Sequence analysis and rarefaction

**Fig 2:**
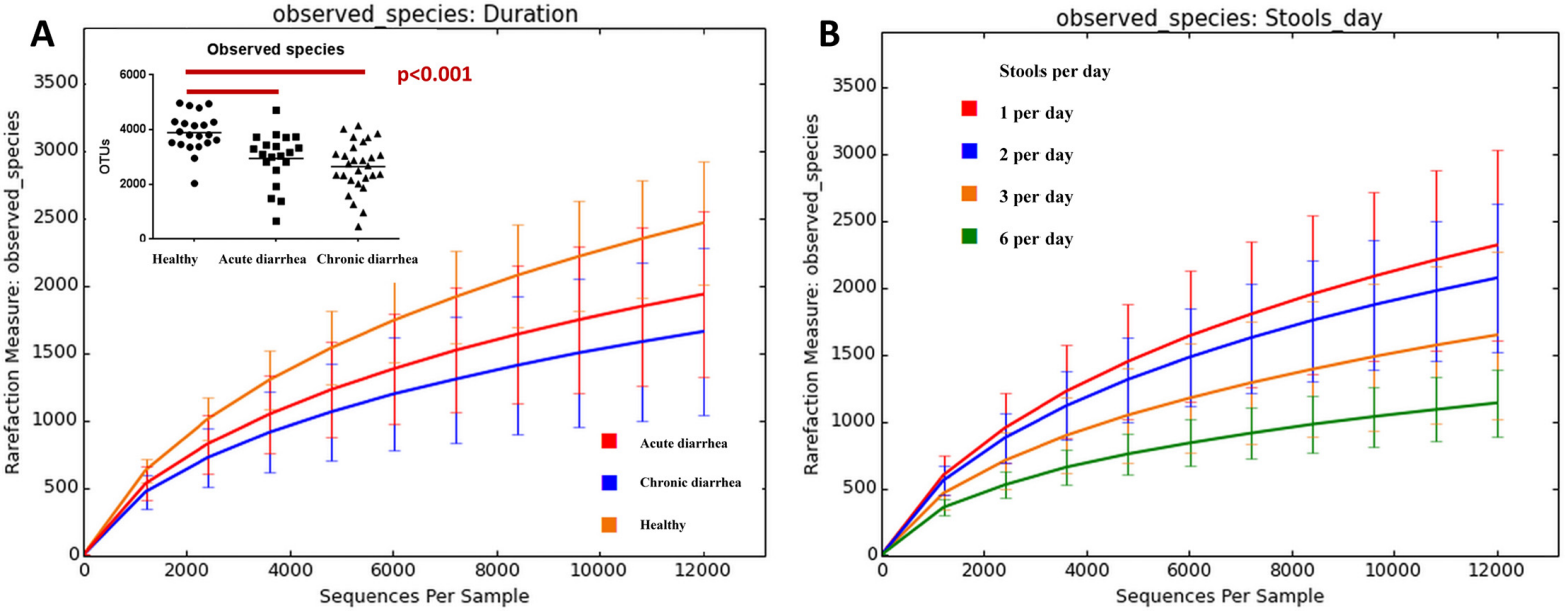
Rarefaction analysis of 16S rRNA gene sequences obtained from feline fecal samples. Lines represent the mean and error bars represent standard deviations. The analysis was performed on a randomly selected subset of 12,000 sequences per sample. (A) Analysis based on duration of diarrhea. (B) Analysis based on the number of stools per day.

The sequence analysis yielded 2,748,939 quality sequences for all analyzed samples (n = 69, mean ± SD = 39,752, ± 12,006). Alpha diversity, as described by species richness, Chao 1, and Shannon diversity index, was significantly decreased in diarrheic cats. Species richness, as defined by the number of observed species, was significantly decreased in cats with acute diarrhea (mean ± SD: 2951 ± 963) and chronic diarrhea (2649 ± 913) when compared to healthy cats (3900 ± 709) (p<0.0001; Fig 2). Cats with more frequent stools per day also had a more significant decrease in species richness (p<0.05; Fig 2). The Chao 1 was significantly decreased in cats with acute diarrhea (p < 0.01; mean ± SD: 5576 ± 2019) and chronic diarrhea (p<0.001; 4890 ± 1898) when compared to healthy cats (7639 ± 1600). The Shannon diversity index was also significantly decreased in cats with acute diarrhea (p<0.05) and cats with chronic diarrhea (p<0.001).

### Microbial communities

**Fig 3:**
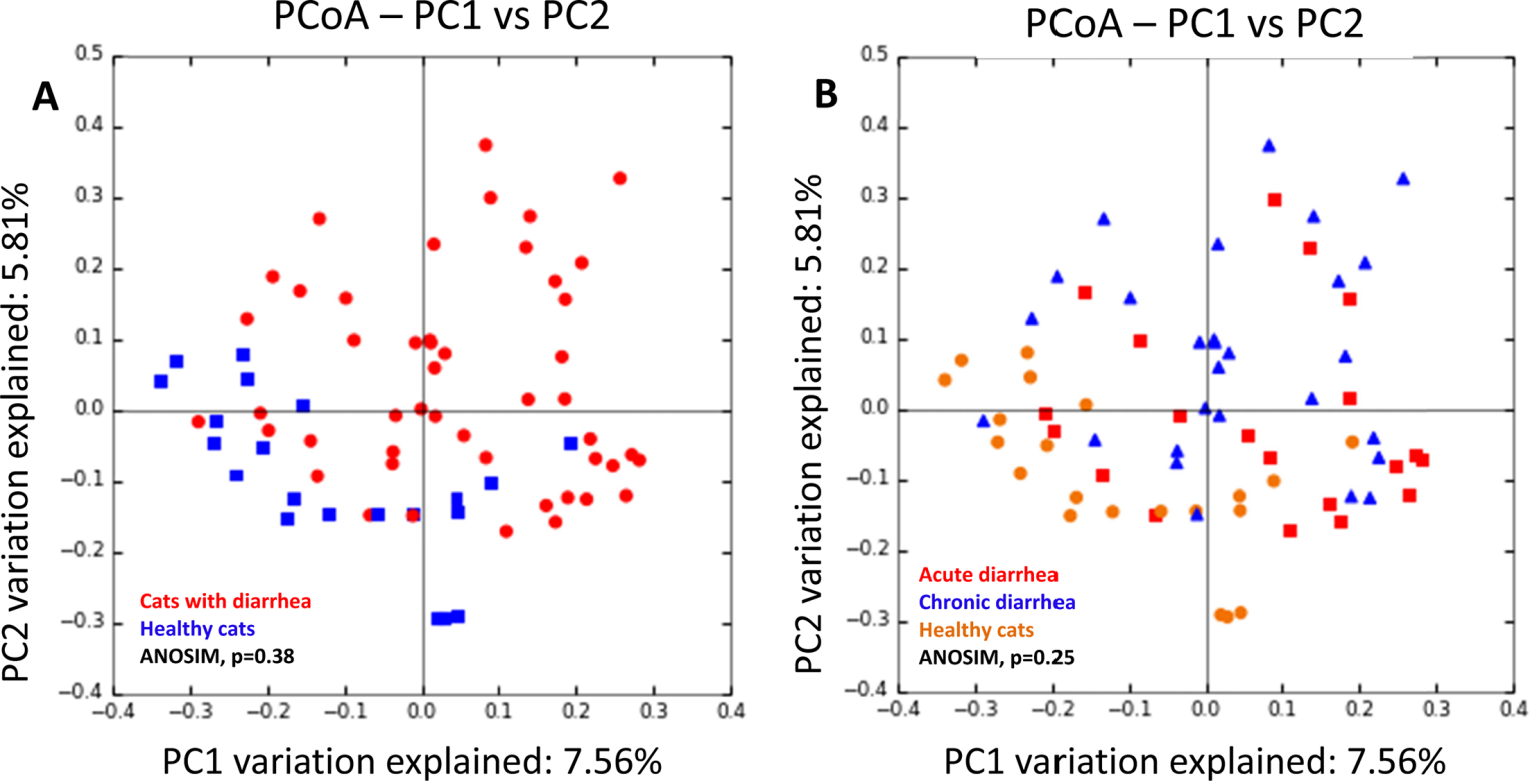
Principal Coordinate Analysis (PCoA) of unweighted UniFrac distances of 16S rRNA genes.

**Fig 4:**
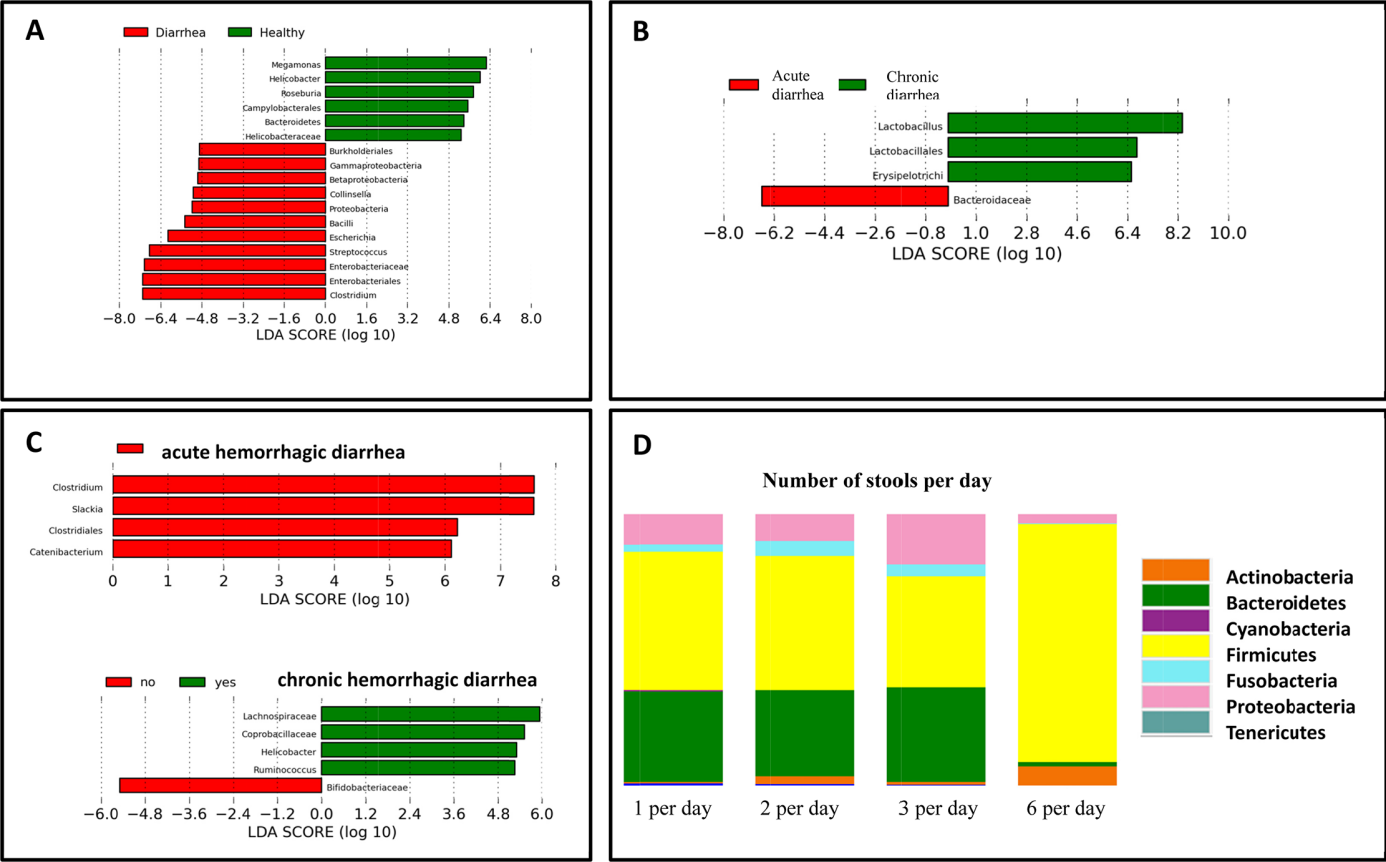
Differentially abundant bacterial groups. Results in A through C display LDA scores based on LEfSe and illustrate which bacterial groups are associated with disease type. (A) All healthy vs all diseased cats; (B) Cats with acute diarrhea vs. cats with chronic diarrhea; (C) Cats with acute hemorrhagic vs. chronic hemorrhagic diarrhea; (D) Changes in bacterial phyla based on the number of stools per day.

Although a strong trend was observed that healthy cats formed a cluster when compared to all cats with diarrhea, this was not significant based on ANOSIM of unweighted Unifrac distances (p = 0.38; Fig 3). No significant differences in microbial communities were observed when the acute and chronic diarrhea groups were analyzed separately (ANOSIM, p = 0.25; Fig 3). However, when individual bacterial groups were analyzed based on LDA effect size (LEfSe) (Fig 4) or Kruskal Wallis tests (S1 Table), several bacterial taxa were identified as being significantly different among the groups. Cats with diarrhea had significantly increased bacterial populations belonging to members of the phylum Proteobacteria (i.e., Gamma-, and Beta-Proteobacteria) and the phylum Firmicutes (class Bacilli and genus *Clostridium*). In contrast, healthy cats had decreases in the class Delta-Proteobacteria, the family Bacteroidaceae, and the genera *Roseburia* and *Megamonas*.

**Fig 5:**
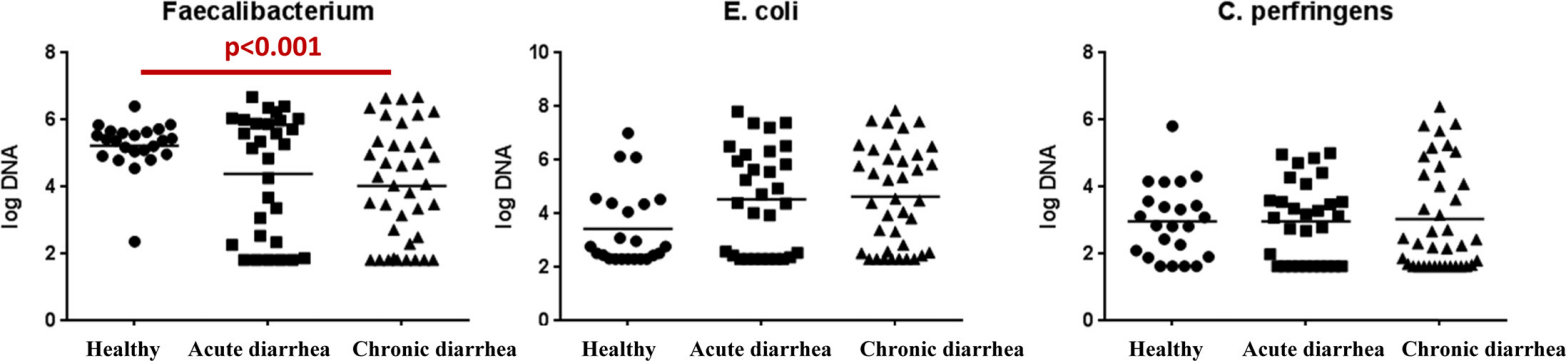
Quantitative PCR results.

Only few altered bacterial groups were observed when feces from cats with acute vs. chronic diarrhea (Fig 4B) were analyzed by LEfSe. In acute diarrhea, the family Bacteroidaceae was increased, while class Erysipelotrichi and genus *Lactobacillus* were increased in chronic diarrhea. The qPCR revealed that *Faecalibacterium* spp. were significantly decreased in cats with chronic diarrhea (p < 0.001; Fig 5) when compared to healthy cats. The abundance of *C*. *perfringens* and *E*. *coli* was not significantly different among the cat groups (Fig 5), although there was a trend for *E*. *coli* to increase in acute diarrhea (p = 0.081).

Significant changes in proportions of bacterial taxa were also observed in acute hemorrhagic vs. chronic hemorrhagic diarrhea (Fig 4C). In acute hemorrhagic diarrhea, increases in proportions of Clostridiales and especially genus *Clostridium* were observed. The subanalysis of the qPCR data suggested an increase in *C*. *perfringens* in hemorrhagic diarrhea ns. non-hemorrhagic diarrhea (p = 0.052). Chronic hemorrhagic diarrhea was associated with the families Lachnospiraceae and Coprobacillaceae, and the genera *Helicobacter* and *Ruminococcus*.

Changes in bacterial phyla were also observed when the data was stratified based on the number of stools per day, with significant decreases in Bacteroidetes in cats with 6 or more stools per day (p < 0.05; Fig 4D).

### Functional genes

**Fig 6:**
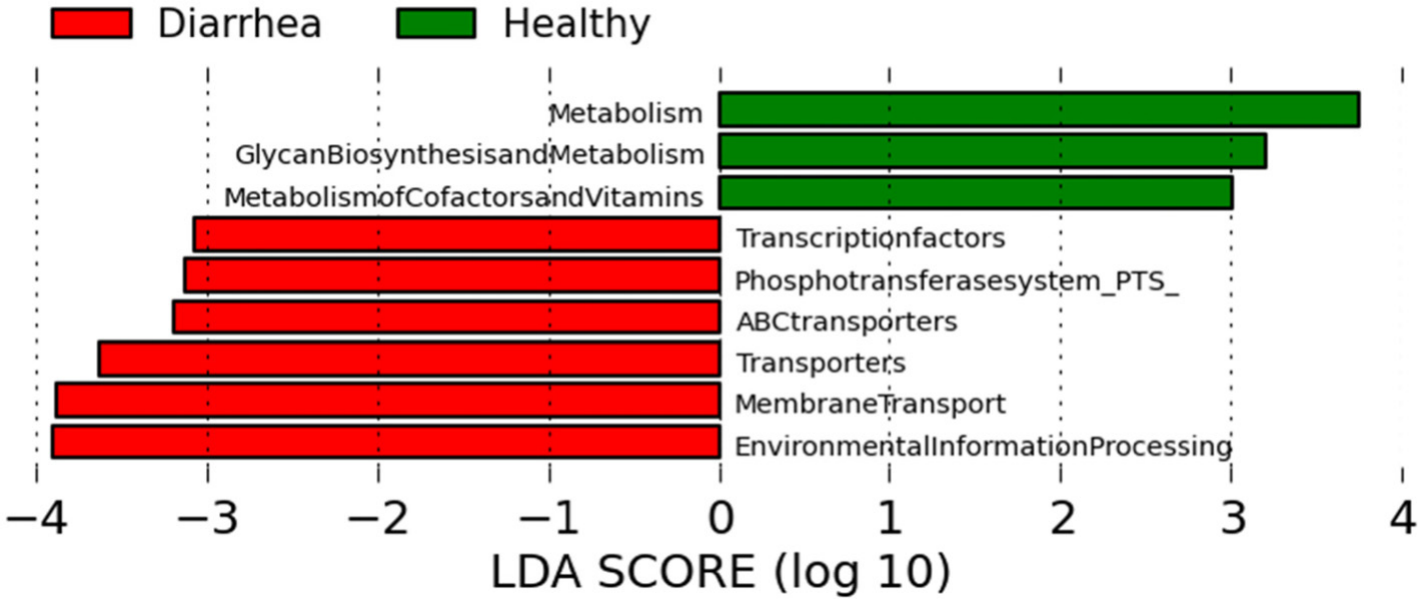
Predicted functional composition of metagenomes based on 16S rRNA gene sequencing data. LEfSe based on the PICRUSt data set revealed differentially enriched bacterial functions associated either with healthy (green) or diseased cats (red).

Several significant differences in the percentage of KEGG orthologs belonging to functional gene categories were identified among all groups of cats after correcting for multiple comparisons (S2 Table) and LEfSe analysis (LDA score > 4; Fig 6). Increased in diarrhea were genes for phosphotransferase system (PTS; p = 0.053), transcription factors (p = 0.028), epithelial cell signaling (p = 0.017), lysine degradation (p = 0.024), tryptophan metabolism (p = 0.034), glycerolipid metabolism (p = 0.013), biodegradation of xenobiotics (p = 0.044), caprolactam degradation (p = 0.025), dioxin degradation (p = 0.008), and xylene degradation (p = 0.006). In contrast, decreased in diarrhea were genes for RNA degradation (p = 0.021), biosynthesis of secondary metabolites (p = 0.047), and biotin metabolism (p = 0.0332). Based on LEfSe, cats with acute hemorrhagic diarrhea had significant increases in genes for retinol metabolism, drug metabolism (cytochrome P450), xenobiotics metabolism, and degradation of toluene. In contrast, cats with acute non-hemorrhagic diarrhea had increases in cysteine and methionine metabolism (LDA > 2).

## Discussion

Various recent studies have described the feline gastrointestinal microbiome in health [21,22] and in response to dietary modifications [6,11,23]. However, limited information is available about changes in the fecal microbiomes that occur in acute or chronic gastrointestinal diseases of cats. Most studies evaluating bacteria in the GI tract of cats with intestinal disease utilized methods targeting specific bacterial groups using FISH. One study evaluated the mucosa-adherent bacteria in the small intestine of cats with IBD and reported an increase in Enterobacteriaceae, and this increase was also associated with the severity of histological inflammation [7]. Fecal samples of cats with IBD had decreased FISH counts for total bacteria, *Bacteroides* spp., and *Bifidobacterium* spp., and increased counts of *Desulfovibrio* spp. compared to healthy cats [13]. *Desulfovibrio* spp. are sulfate-reducing bacteria that produce hydrogen sulfides, and have been suggested to be involved in the pathogenesis of feline IBD [13]. However, a follow-up study did not identify any significant differences in FISH counts between cats with IBD and controls, although the same bacterial groups were targeted [12]. Our study also did not reveal any differences in *Desulfovibrio* spp. among the groups. A recent study utilized 454-pyrosequencing of 16S rRNA genes to describe changes in fecal microbiota in cats with chronic diarrhea and their response to dietary modifications [14]. Several bacterial groups correlated with improved fecal scores after therapeutic response to diet. Those included the family Enterobacteriaceae, unidentified genera within the order Clostridiales and family Lachnospiraceae, and various species such as *Slackia* spp., *Campylobacter upsaliensis*, and *Collinsella* spp. [14]. However, no comparison in the fecal microbiota between healthy and diarrheic cats was performed in this reported study [14].

In the current study, we compared the fecal microbiome between healthy cats and cats with acute or chronic diarrhea. Our results revealed significant decreases in species richness and specific bacterial taxa, similar to previous observations in humans and dogs with GI disease [24,25]. Principal coordinates analysis (PCoA) plots revealed no significant differences in microbiome composition among the 3 groups based on statistical analysis. However, trends were observed for the healthy cats to cluster more closely when compared to the diarrheic cats, suggesting that there are differences in overall microbiota structure between healthy and diseased animals. This was confirmed through statistical analysis of specific taxa as well as qPCR data. Generally we observed similar broad trends that have been reported in GI disease of humans and dogs [1,24,25], with increases in members of Gamma-, and Beta-Proteobacteria and decreases in Bacteroidetes. Similarly as in other animal species, we also observed changes within Bacilli and Clostridium, and decreases in members of *Clostridium* clusters XIV and IV. While we observed significant increases in the genus *Clostridium* in the cats with diarrhea, the qPCR data for *C*. *perfringens* did not show significant increases in this species. This is somewhat different compared to dogs, where increases in *C*. *perfringens* are commonly observed in both acute and chronic diarrhea [1,3,26]. *Clostridium perfringens* has been suggested to serve primarily as a marker of intestinal dysbiosis in dogs, and its role as primary enteropathogen remains unclear [1,27]. Our data would suggest that *C*. *perfringens* does not play a significant role in GI disease of cats. Another bacterial species often implicated in GI disease is *E*. *coli* [1,9,25]. There were significant increases in *Escherichia* observed in the sequence data, and a trend was noted for an increase in *E*. *coli* in acute diarrhea based on qPCR data. Further studies evaluating the role of *E*. *coli* either as marker of dysbiosis or as enteropathogen contributing to feline GI disease are warranted.

The decreases in *Clostridium* clusters XIV and IV are consistent with data observed in humans and dogs. *Faecalibacterium* is a bacterial genus that has been frequently reported to be decreased in GI inflammation and is becoming recognized as an important marker for GI health and important for maintaining GI health [25,28]. Increases in fecal abundances of *Faecalibacterium* have also been associated with improvement of clinical disease severity in dogs with IBD [3,19] and this would be worthwhile evaluating in cats in longitudinal studies.

Of importance is that many bacterial groups within *Clostridium* clusters XIV and IV are considered important producers of short-chain fatty acids (SCFA) and other metabolites [29]. Therefore, functional studies (e.g., metabolomics or metagenomics) will be important in the future to understand whether these microbial changes lead to functional deficiencies in the feline gut. Several studies in humans with Crohn’s disease or mouse models of experimental colitis have identified altered microbiome function using either a true metagenomic approach by DNA shotgun sequencing or by PICRUSt analysis of 16S rRNA genes [30–33]. In this study, we used 16S rRNA gene profiles to infer putative metagenomes based on the software PICRUSt [15] and identified several functional gene categories as differentially expressed between healthy cats and cats with diarrhea (Fig 6 and S2 Table). Increased abundance of genes belonging to the phosphotransferase system (PTS) were associated with diarrhea, suggesting altered carbohydrate metabolism of gut bacteria in GI disease, similar to data observed in humans with Crohn’s disease [30]. The gut microbiome of cats with diarrhea also had decreased abundances of genes responsible for metabolism of various vitamins (e.g., biotin), amino acids (e.g., tryptophan, lysine, cysteine, and methionine) and various co-factors and glycan biosynthesis. The changes in amino acids are similar as observed in dogs with chronic diarrhea due to idiopathic IBD [25] and humans with Crohn’s disease [31], suggesting that amino acid dysmetabolism may be an important feature of chronic GI disease. Another important altered pathway in cats with diarrhea was the metabolism of xenobiotics, which has also been observed previously in GI inflammation [31]. All these changes suggest a severe dysfunction in gut microbiota that manifest itself on various functional levels. Therefore, further investigations into the functional capacity by direct measuring of metabolites will be an important next step for better understanding of the pathophysiology of GI disease.

There were several limitations of this study. In this study, we utilized Ion Torrent technology for sequencing of PCR amplicons, and it has been recently described that this technique has an increased error rate for 16S rRNA amplicons compared to traditional 454-pyrosequencing or Illumina next-generation sequencing [34], although this technique has been used successfully in several microbiota studies [35,36]. However, because we used the same protocol across all diseased groups, it is unlikely that the error rate would be different across animal groups. PICRUSt is solely a predictor of function potential, and a true metagenomics approach may yield more in depth resolution of metagenomic changes in cats with acute or chronic diarrhea. We observed significant differences in age and BCS between the healthy cats and the cats with diarrhea. Cats with chronic diarrhea were significantly older compared to healthy cats. Juvenile kittens of less than 1 year of age were reported as a confounding factor for gut microbiome analysis, and therefore we excluded all cats in this age group from analysis [37]. Less is known about the microbial changes in older cats. A recent study described decreased abundances in *Faecalibacterium* in cats older than 10 years vs. cats younger than 10 years, however, no other bacterial groups were altered [38]. Also another study did not observe obvious age effects in geriatric cats [39]. Furthermore, as Fig 1 illustrates, despite significant differences, there was a wide age distribution among all groups, making it less likely that age differences were a major confounding factor in this study. The BCS is used to assed whether animals were under- or overweight. The BCS distribution was also wide among groups and it is unlikely that the here observed differences in bacterial groups were solely due to BCS differences, as also only minor differences in few bacterial genera were observed in dogs with varying BCS [40]. The reason for the observed differences in ages and BCS reflect the population of clinical patients that were evaluated in this study and it is difficult to completely match patient groups for age and other parameters. It would also have been of interest to correlate the presence of selected enteropathogens with specific microbiome patterns; however, there were too few animals in each group to perform meaningful statistical comparisons. Further studies are warranted to evaluate other members of the microbiome such as fungi and viruses to better understand the microbial dynamics in diarrhea [41].

## Conclusions

In conclusion, results of this study revealed a bacterial dysbiosis in fecal samples of cats with acute and chronic diarrhea. Species richness was significantly decreased and various bacterial taxa were differentially altered. Initial data would suggest that these changes are associated with functional alterations in the feline microbiome and this warrants further investigations.

## Supporting Information

S1 Table Differences in bacterial taxa on various phylogenetic levels between healthy cats and cats with acute or chronic diarrhea. (DOCX)

S2 Table Percentages of KEGG orthologs that belong to functional categories at levels 1, 2, and 3. (DOCX)
