# Supplementary material for: The Fecal Microbiome in Cats with Diarrhea
Source: PLoS One. 2015 May 19;10(5):e0127378. doi: 10.1371/journal.pone.0127378 (PMC4437779; doi:10.1371/journal.pone.0127378)
Supplement: S1 Table — (DOCX) [file pone.0127378.s001.docx]

| **S1 Table. Differences in bacterial taxa on various phylogenetic levels between healthy cats and cats with acute or chronic diarrhea.** | | | | | | | |  |  |  |
| --- | --- | --- | --- | --- | --- | --- | --- | --- | --- | --- |
|  |  |  |  |  |  |  |  |  |  |  |
|  | **Range (Minimum %-Maximum %) and Medians (%)** | | | | | | |  |  |  |
|  | Range | | |  | Median | | |  |  | |
|  | Healthy | Acute Diarrhea | Chronic Diarrhea |  | Healthy | Acute Diarrhea | Chronic Diarrhea |  | Kruskal Wallace P-value | **Kruskal Wallace adjusted P-value** |
| Actinobacteria | 0-6.66 | 0-16.1 | 0-26.9 |  | 0.11 | 0.04 | 0.09 |  | 0.3545 | 0.7090 |
| Bacteroidetes | 14.4-73.0 | 0.08-62.7 | 0-73.8 |  | 33.34 | 27.83 | 21.38 |  | 0.0796 | 0.2388 |
| Firmicutes | 6.98-81.8 | 17.9-90.5 | 2-99.8 |  | 50.58 | 56.31 | 51.41 |  | 0.7407 | 0.6349 |
| Fusobacteria | 0-16.4 | 0-30.4 | 0-37.9 |  | 1.15 | 1.12 | 0.25 |  | 0.3689 | 0.4427 |
| Proteobacteria | 0.26-27.9 | 0.07-45.0 | 0.02-83.4 |  | 4.79 | 6.90 | 5.72 |  | 0.6473 | 0.6473 |
| Actinobacteria | 0-26.9 | 0-16.1 | 0-26.9 |  | 0.09 | 0.04 | 0.08 |  | 0.2439 | 0.2627 |
| Bacteroidetes_Other (class) | 0-0.22 | 0-0.21 | 0-0.06 |  | 0.04^a^ | 0.01^a,b^ | 0.01^b^ |  | 0.0002 | **0.0007** |
| Bacteroidia | 0-73.8 | 0.08-62.6 | 0-73.8 |  | 30.70 | 27.82 | 18.59 |  | 0.0796 | 0.1194 |
| Firmicutes_Other (class) | 0-1.95 | 0.02-1.91 | 0-1.27 |  | 0.52^a^ | 0.39^a,b^ | 0.34^b^ |  | 0.0185 | **0.0432** |
| Bacilli | 0-97.5 | 0-81.9 | 0.00-97.5 |  | 0.22^a^ | 2.14^b^ | 1.78^b^ |  | 0.0001 | **0.0012** |
| Clostridia | 0-78.0 | 0.94-80.3 | 0-71.5 |  | 38.87 | 38.56 | 38.31 |  | 0.0852 | 0.1136 |
| Erysipelotrichi | 0-43.9 | 0.00-32.1 | 0-43.9 |  | 1.88 | 0.75 | 1.20 |  | 0.5170 | 0.5170 |
| Fusobacteria | 0-24.5 | 0-30.4 | 0-24.5 |  | 1.07 | 1.12 | 0.99 |  | 0.3689 | 0.4024 |
| Proteobacteria_Other (class) | 0-4.9 | 0-0.38 | 0-4.9 |  | 0.06^a^ | 0.02^b^ | 0.02^b^ |  | 0.0072 | **0.0202** |
| Betaproteobacteria | 0-20.4 | 0-5.37 | 0-20.4 |  | 0.31 | 0.06 | 0.11 |  | 0.0683 | 0.1171 |
| Deltaproteobacteria | 0-3.01 | 0-4.14 | 0-3.01 |  | 0.02 | 0.01 | 0.00 |  | 0.1656 | 0.1987 |
| Epsilonproteobacteria | 0-27.0 | 0-20.5 | 0-2.98 |  | 0.7^a^ | 0.05^b^ | 0.03^b^ |  | 0.0001 | **0.0006** |
| Gammaproteobacteria | 0-75.8 | 0-44.6 | 0.00-75.8 |  | 0.66^a^ | 3.77^b^ | 1.61^a,b^ |  | 0.0069 | **0.0166** |
| Actinomycetales | 0-0.34 | 0-16.1 | 0-4.60 |  | 0.00 | 0.01 | 0.00 |  | 0.0971 | 0.1665 |
| Bifidobacteriales | 0-6.66 | 0-5.5 | 0-26.9 |  | 0.10 | 0.00 | 0.07 |  | 0.1668 | 0.2502 |
| Bacteroidales | 14.3-73.0 | 0.08-62.6 | 0-73.8 |  | 33.31 | 27.82 | 21.36 |  | 0.0796 | 0.1737 |
| Bacilli_Other (order) | 0-0.18 | 0-0.66 | 0-1.51 |  | 0.01 | 0.02 | 0.02 |  | 0.2320 | 0.3093 |
| Bacillales | 0-0.54 | 0-67.3 | 0-5.66 |  | 0.00 | 0.00 | 0.00 |  | 0.8378 | 0.9140 |
| Lactobacillales | 0-0.66 | 0-81.9 | 0.00-97.4 |  | 0.05^a^ | 0.54^b^ | 1.51^b^ |  | 0.0001 | **0.0012** |
| Turicibacterales | 0-1.53 | 0-15.2 | 0-12.9 |  | 0.01 | 0.00 | 0.00 |  | 0.9637 | 0.9637 |
| Clostridia_Other (order) | 0.42-6.03 | 0.04-14.2 | 0-6.95 |  | 1.61 | 1.90 | 1.70 |  | 0.8834 | 0.8834 |
| Clostridia_ | 0-16.3 | 0-3.44 | 0-4.4 |  | 0.21 | 0.10 | 0.09 |  | 0.1666 | 0.2666 |
| Clostridiales | 6.50-71.1 | 0.72-75.0 | 0-67.7 |  | 38.71 | 33.20 | 30.98 |  | 0.0848 | 0.1696 |
| Coriobacteriales | 0-0.78 | 0-3.98 | 0-3.87 |  | 0.03^a^ | 0.13^b^ | 0.11^a,b^ |  | 0.0294 | 0.0706 |
| Erysipelotrichales | 0-42.1 | 0.00-32.1 | 0-43.9 |  | 2.17 | 0.75 | 1.24 |  | 0.5170 | 0.6531 |
| Fusobacteriales | 0-16.4 | 0-30.4 | 0-37.9 |  | 1.15 | 1.12 | 0.25 |  | 0.3689 | 0.5208 |
| Betaproteobacteria_Other (order) | 0-0.55 | 0-0.47 | 0-2.58 |  | 0.03^a^ | 0^a,b^ | 0.01^b^ |  | 0.0099 | **0.0297** |
| Burkholderiales | 0.00-2.02 | 0-5.22 | 0-17.8 |  | 0.26 | 0.06 | 0.12 |  | 0.0859 | 0.1586 |
| Desulfovibrionales | 0-1.04 | 0-4.14 | 0-7.78 |  | 0.04 | 0.01 | 0.00 |  | 0.1317 | 0.2107 |
| Campylobacterales | 0.01-27.0 | 0-20.5 | 0-2.98 |  | 1.59^a^ | 0.05^b^ | 0.02^b^ |  | 0.0001 | **0.0008** |
| Gammaproteobacteria_Other (order) | 0-0.48 | 0-1.36 | 0-3.53 |  | 0.01 | 0.04 | 0.03 |  | 0.3211 | 0.4056 |
| Aeromonadales | 0-4.72 | 0-15.4 | 0-39.7 |  | 0.02 | 0.11 | 0.02 |  | 0.9374 | 0.9782 |
| Enterobacteriales | 0-7.99 | 0-44.1 | 0-77.3 |  | 0.00^a^ | 0.36^b^ | 0.27^b^ |  | 0.0001 | **0.0006** |
| Bifidobacteriaceae | 0-6.65 | 0-5.48 | 0-26.9 |  | 0.10 | 0.00 | 0.07 |  | 0.1539 | 0.2280 |
| Bacteroidales_Other (family) | 0.02-1.13 | 0-4.17 | 0-3.02 |  | 0.17 | 0.08 | 0.04 |  | 0.0232 | 0.0580 |
| Bacteroidaceae | 4.12-73 | 0.02-51.9 | 0-38.1 |  | 10.94^a^ | 8.63^a,b^ | 4.43^b^ |  | 0.0108 | **0.0360** |
| Porphyromonadaceae | 0-2.82 | 0-2.3 | 0-20.9 |  | 0.54 | 0.21 | 0.07 |  | 0.1544 | 0.2206 |
| Prevotellaceae | 0-47.4 | 0-42.4 | 0-69.5 |  | 7.95 | 0.87 | 4.93 |  | 0.4432 | 0.5719 |
| Rikenellaceae | 0-0.85 | 0-1.21 | 0-0.91 |  | 0.02^a^ | 0.01^a,b^ | 0.00^b^ |  | 0.0158 | **0.0421** |
| [Odoribacteraceae] | 0-13.4 | 0-12.5 | 0-5.72 |  | 0.01^a,b^ | 0.08^a^ | 0.00^b^ |  | 0.0277 | 0.0554 |
| [Paraprevotellaceae] | 0-2.64 | 0-15.5 | 0-14.0 |  | 0.00 | 0.01 | 0.00 |  | 0.6628 | 0.7575 |
| Lactobacillales_Other (family) | 0-0.01 | 0-0.36 | 0-0.32 |  | 0.00^a^ | 0.00^a^ | 0.01^b^ |  | 0.0091 | **0.0280** |
| Enterococcaceae | 0-0.66 | 0-6.87 | 0-16.5 |  | 0.01 | 0.03 | 0.02 |  | 0.5509 | 0.6481 |
| Lactobacillaceae | 0-0.57 | 0-81.2 | 0-80.6 |  | 0.00^a^ | 0.00^b^ | 0.023^a,b^ |  | 0.0183 | **0.0458** |
| Streptococcaceae | 0-0.08 | 0-12.4 | 0-63.2 |  | 0.00^a^ | 0.02^b^ | 0.09^b^ |  | 0.0001 | **0.0020** |
| Turicibacteraceae | 0-1.53 | 0-15.2 | 0-12.9 |  | 0.01 | 0.00 | 0.00 |  | 0.9637 | 0.9637 |
| Clostridiales_Other (family) | 0.06-2.58 | 0.01-3.36 | 0-3.58 |  | 1.14 | 0.81 | 0.56 |  | 0.0453 | 0.0906 |
| Clostridiaceae | 0-10.8 | 0.03-67.9 | 0-48.5 |  | 0.66 | 2.23 | 1.23 |  | 0.0994 | 0.1590 |
| Lachnospiraceae | 1.94-36.5 | 0.15-44.6 | 0-51.1 |  | 9.06 | 10.46 | 10.18 |  | 0.9034 | 0.9766 |
| Peptococcaceae | 0-1.09 | 0-2.54 | 0-1.87 |  | 0.07 | 0.02 | 0.00 |  | 0.1591 | 0.2194 |
| Peptostreptococcaceae | 0-0.12 | 0-4.73 | 0-4.57 |  | 0.00^a^ | 0.01^a,b^ | 0.07^b^ |  | 0.0018 | **0.0090** |
| Ruminococcaceae | 0-36.9 | 0.02-25.2 | 0-26.6 |  | 9.01^a^ | 4.57^a,b^ | 1.89^b^ |  | 0.0007 | **0.0047** |
| Veillonellaceae | 0.35-54.1 | 0-50.1 | 0-54.2 |  | 9.14^a^ | 4.31^b^ | 3.87^b^ |  | 0.0360 | 0.0655 |
| Coriobacteriaceae | 0-0.78 | 0-3.98 | 0-3.87 |  | 0.03^a^ | 0.13^b^ | 0.11^a,b^ |  | 0.0302 | 0.0575 |
| Erysipelotrichaceae | 0-32.0 | 0-32.0 | 0-36.9 |  | 0.55 | 0.52 | 0.61 |  | 0.9182 | 0.9665 |
| [Coprobacillaceae] | 0-12.9 | 0-3.61 | 0-15.8 |  | 0.87^a^ | 0.07^b^ | 0.25^a,b^ |  | 0.0154 | **0.0474** |
| Fusobacteriaceae | 0-16.4 | 0-30.4 | 0-37.9 |  | 1.15 | 1.12 | 0.25 |  | 0.3689 | 0.4919 |
| Burkholderiales_Other (family) | 0-0.77 | 0-0.55 | 0-2.02 |  | 0.10^a^ | 0.01^b^ | 0.02^b^ |  | 0.0007 | **0.0047** |
| Alcaligenaceae | 0.00-1.34 | 0-4.70 | 0-16.3 |  | 0.21 | 0.04 | 0.09 |  | 0.0787 | 0.1312 |
| Desulfovibrionaceae | 0-1.02 | 0-4.11 | 0-7.76 |  | 0.03 | 0.01 | 0.00 |  | 0.1104 | 0.1698 |
| Campylobacteraceae | 0-7.39 | 0-7.49 | 0-1.08 |  | 0.20^a^ | 0.01^b^ | 0.01^b^ |  | 0.0050 | **0.0182** |
| Helicobacteraceae | 0-25.5 | 0-19.6 | 0-2.74 |  | 0.35^a^ | 0.00^b^ | 0.00^b^ |  | 0.0002 | **0.0016** |
| Succinivibrionaceae | 0-4.72 | 0-15.4 | 0-39.7 |  | 0.02 | 0.11 | 0.02 |  | 0.9374 | 0.9614 |
| Enterobacteriaceae | 0-8.41 | 0-44.1 | 0-77.3 |  | 0.00^a^ | 0.36^b^ | 0.27^b^ |  | 0.0001 | **0.0013** |
| Bifidobacteriaceae_Other (genus) | 0-0.15 | 0-0.04 | 0-0.19 |  | 0.00 | 0.00 | 0.00 |  | 0.3204 | 0.4369 |
| Bifidobacterium | 0-6.6 | 0-5.46 | 0-26.8 |  | 0.10 | 0.00 | 0.07 |  | 0.1576 | 0.2712 |
| Bacteroidaceae_Other (genus) | 0.04-3.25 | 0-3.5 | 0-4.14 |  | 0.41 | 0.25 | 0.09 |  | 0.4432 | 0.5963 |
| Bacteroides | 3.60-72.5 | 0.02-49.4 | 0-34.0 |  | 10.65^a^ | 8.32^a,b^ | 4.31^b^ |  | 0.0124 | **0.0417** |
| Parabacteroides | 0-2.78 | 0-2.3 | 0-20.9 |  | 0.53 | 0.21 | 0.07 |  | 0.1544 | 0.2720 |
| Prevotella | 0-47.4 | 0-42.4 | 0-69.5 |  | 7.95 | 0.87 | 4.93 |  | 0.4432 | 0.5963 |
| Rikenellaceae_ (genus) | 0-0.85 | 0-1.18 | 0-0.9 |  | 0.02 | 0.01 | 0.00 |  | 0.1727 | 0.3084 |
| Odoribacter | 0-13.4 | 0-12.5 | 0-5.6 |  | 0.01 | 0.02 | 0.00 |  | 0.0779 | 0.1601 |
| [Prevotella] | 0-2.64 | 0-15.5 | 0-13.8 |  | 0.00 | 0.00 | 0.00 |  | 0.8019 | 0.8727 |
| Enterococcaceae_Other (genus) | 0-0.01 | 0-0.28 | 0-3.15 |  | 0.00 | 0.00 | 0.00 |  | 0.1772 | 0.3020 |
| Enterococcus | 0-0.65 | 0-6.59 | 0-13.4 |  | 0.01 | 0.02 | 0.02 |  | 0.5935 | 0.6971 |
| Lactobacillus | 0-0.56 | 0-30.4 | 0-80.6 |  | 0.00^a^ | 0.00^a,b^ | 0.02^b^ |  | 0.0364 | 0.0816 |
| Streptococcus | 0-0.08 | 0-12.2 | 0-60.9 |  | 0.00^a^ | 0.01^b^ | 0.09^b^ |  | 0.0001 | **0.0037** |
| Turicibacter | 0-1.53 | 0-15.2 | 0-12.9 |  | 0.01 | 0.00 | 0.00 |  | 0.9637 | 1.0044 |
| Clostridiaceae_Other (genus) | 0-0.40 | 0-6.32 | 0-4.74 |  | 0.05 | 0.04 | 0.06 |  | 0.1748 | 0.2980 |
| Clostridiaceae_(genus) | 0-1.90 | 0-1.64 | 0-1.56 |  | 0.05 | 0.02 | 0.00 |  | 0.4694 | 0.5678 |
| Clostridium | 0-10.8 | 0.01-67.4 | 0-48.4 |  | 0.33 | 1.16 | 0.64 |  | 0.1052 | 0.1996 |
| Lachnospiraceae_Other (genus) | 0.96-22.4 | 0.08-22.8 | 0-29.6 |  | 6.75 | 6.79 | 5.30 |  | 0.7248 | 0.8113 |
| Lachnospiraceae_(genus) | 0-1.04 | 0.01-1.95 | 0-7.64 |  | 0.27 | 0.51 | 0.47 |  | 0.0659 | 0.1373 |
| Blautia | 0-5.45 | 0.01-13.0 | 0-15.1 |  | 1.48 | 1.28 | 1.49 |  | 0.9980 | 0.9980 |
| Coprococcus | 0-0.26 | 0-0.10 | 0-0.98 |  | 0.01^a^ | 0^a,b^ | 0.00^b^ |  | 0.0418 | 0.0910 |
| Dorea | 0-0.28 | 0-0.67 | 0-2.4 |  | 0.01 | 0.01 | 0.01 |  | 0.6928 | 0.7768 |
| Roseburia | 0-10.3 | 0-2.51 | 0-4.78 |  | 0.15^a^ | 0.01^b^ | 0.01^b^ |  | 0.0001 | **0.0025** |
| [Ruminococcus] | 0-2.4 | 0-29.5 | 0-12.6 |  | 0.07 | 0.17 | 0.20 |  | 0.3367 | 0.4792 |
| Peptococcus | 0-1.09 | 0-2.52 | 0-1.87 |  | 0.07 | 0.02 | 0.00 |  | 0.1217 | 0.2197 |
| Peptostreptococcaceae_Other (genus) | 0-0.08 | 0-1.60 | 0-3.3 |  | 0.00^a^ | 0.01^a,b^ | 0.02^b^ |  | 0.0162 | 0.0528 |
| Peptostreptococcaceae_(genus) | 0-0.04 | 0-4.03 | 0-2.79 |  | 0.00 | 0.00 | 0.01 |  | 0.0337 | 0.0936 |
| Ruminococcaceae_Other (genus) | 0-27.7 | 0-13.6 | 0-17.8 |  | 3.99 | 1.65 | 0.74 |  | 0.0496 | 0.1283 |
| Ruminococcaceae_(genus) | 0-16.6 | 0-11.5 | 0-12.7 |  | 2.47^a^ | 0.88^a,b^ | 0.34^b^ |  | 0.0015 | **0.0113** |
| Faecalibacterium | 0-2.99 | 0-7.09 | 0-13.6 |  | 0.19 | 0.28 | 0.01 |  | 0.0833 | 0.1622 |
| Oscillospira | 0-2.93 | 0-1.92 | 0-1.9 |  | 0.74^a^ | 0.35^a,b^ | 0.18^b^ |  | 0.0024 | **0.0118** |
| Ruminococcus | 0-9.58 | 0-7.06 | 0-1.38 |  | 0.31^a^ | 0.09^a,b^ | 0.05^b^ |  | 0.0351 | 0.0812 |
| Veillonellaceae_Other (genus) | 0.12-12.7 | 0-15.8 | 0-6.76 |  | 2.17^a^ | 0.88^a,b^ | 0.54^b^ |  | 0.0052 | **0.0260** |
| Veillonellaceae_(genus) | 0-1.05 | 0-0.13 | 0-1.10 |  | 0.02 | 0.01 | 0.00 |  | 0.1064 | 0.2100 |
| Acidaminococcus | 0-10.2 | 0-7.78 | 0-22.8 |  | 0.04^a^ | 0a,b | 0.00^b^ |  | 0.0191 | 0.0523 |
| Megamonas | 0-29.6 | 0-12.9 | 0-50.9 |  | 3.13^a^ | 0.18^b^ | 0.14^b^ |  | 0.0001 | **0.0015** |
| Megasphaera | 0-21.3 | 0-17.1 | 0-24.0 |  | 0.75 | 0.24 | 0.04 |  | 0.4006 | 0.5490 |
| Phascolarctobacterium | 0-1.64 | 0-2.54 | 0-2.52 |  | 0.03 | 0.00 | 0.00 |  | 0.1906 | 0.3001 |
| Coriobacteriaceae_Other (genus) | 0-0.43 | 0-2.66 | 0-2.11 |  | 0.01 | 0.00 | 0.00 |  | 0.8944 | 0.9065 |
| Collinsella | 0-0.71 | 0-1.38 | 0-2.27 |  | 0.01^a^ | 0.09^b^ | 0.04^b^ |  | 0.0003 | **0.0022** |
| Erysipelotrichaceae_Other (genus) | 0-0.22 | 0-0.42 | 0-0.69 |  | 0.00 | 0.00 | 0.00 |  | 0.5600 | 0.6885 |
| Bulleidia | 0-7.42 | 0-13.0 | 0-36.2 |  | 0.00 | 0.00 | 0.00 |  | 0.9310 | 0.9842 |
| [Eubacterium] | 0-31.9 | 0-29.8 | 0-25.3 |  | 0.45 | 0.35 | 0.21 |  | 0.7649 | 0.8448 |
| [Coprobacillaceae]_Other (genus) | 0-0.23 | 0-0.07 | 0-0.78 |  | 0.01^a^ | 0.00^b^ | 0^a,b^ |  | 0.0014 | **0.0263** |
| [Coprobacillaceae]_(genus) | 0-1.68 | 0-0.14 | 0-5.01 |  | 0.01 | 0.00 | 0.00 |  | 0.0109 | 0.0629 |
| Catenibacterium | 0-12.9 | 0-3.54 | 0-15.3 |  | 0.70 | 0.03 | 0.03 |  | 0.1196 | 0.2213 |
| Fusobacteriaceae_Other (genus) | 0-1.12 | 0-2.12 | 0-3.2 |  | 0.09 | 0.07 | 0.01 |  | 0.0946 | 0.1971 |
| Fusobacteriaceae_(genus) | 0-0.75 | 0-28.1 | 0-22.9 |  | 0.00 | 0.00 | 0.00 |  | 0.1198 | 0.2304 |
| J2-29 | 0-15.2 | 0-15.5 | 0-36.6 |  | 0.97 | 0.56 | 0.06 |  | 0.1733 | 0.2850 |
| Alcaligenaceae_Other (genus) | 0-0.09 | 0-0.04 | 0-2.20 |  | 0.01 | 0.00 | 0.00 |  | 0.0361 | 0.0967 |
| Sutterella | 0.00-1.25 | 0-4.70 | 0-14.1 |  | 0.19 | 0.04 | 0.08 |  | 0.0781 | 0.1562 |
| Desulfovibrio | 0-0.96 | 0-4.05 | 0-7.46 |  | 0.01 | 0.00 | 0.00 |  | 0.2779 | 0.4197 |
| Campylobacter | 0-7.39 | 0-7.49 | 0-1.08 |  | 0.20^a^ | 0.01^b^ | 0.01^b^ |  | 0.0050 | **0.0195** |
| Helicobacteraceae_Other (genus) | 0-3.03 | 0-3.73 | 0-0.21 |  | 0.05^a^ | 0.00^b^ | 0.00^b^ |  | 0.0013 | **0.0108** |
| Helicobacter | 0-24.0 | 0-15.9 | 0-2.72 |  | 0.29^a^ | 0.00^b^ | 0.00^b^ |  | 0.0003 | **0.0020** |
| Succinivibrionaceae_Other (genus) | 0-0.23 | 0-1.15 | 0-0.8 |  | 0.00 | 0.00 | 0.00 |  | 0.5563 | 0.6954 |
| Succinivibrionaceae_(genus) | 0-2.34 | 0-2.33 | 0-0.42 |  | 0.00 | 0.00 | 0.00 |  | 0.2581 | 0.4033 |
| Anaerobiospirillum | 0-2.72 | 0-15.3 | 0-39.5 |  | 0.00 | 0.02 | 0.00 |  | 0.3852 | 0.5378 |
| Enterobacteriaceae_Other (genus) | 0-7.32 | 0-37.9 | 0-65.3 |  | 0.00^a^ | 0.31^b^ | 0.24^b^ |  | 0.0003 | **0.0028** |
| Escherichia | 0-1.08 | 0-6.15 | 0-11.7 |  | 0.00^a^ | 0.05^b^ | 0.05^b^ |  | 0.0011 | **0.0063** |
| *Medians not sharing a common superscript are significantly different (p<0.05 based on Dunn's multiple comparisons test).* | | | | | | |  |  |  |  |
