# Supplementary material for: The Fecal Microbiome in Cats with Diarrhea
Source: PLoS One. 2015 May 19;10(5):e0127378. doi: 10.1371/journal.pone.0127378 (PMC4437779; doi:10.1371/journal.pone.0127378)
Supplement: S2 Table — (DOCX) [file pone.0127378.s002.docx]

| **S2 Table. Percentages of KEGG orthologs that belong to functional categories at levels 1, 2, and 3.** | | | | | | | | | | |
| --- | --- | --- | --- | --- | --- | --- | --- | --- | --- | --- |
|  |  |  |  |  |  |  |  |  |  |  |
| **KEGG Ortholog** | **Range (Minimum %-Maximum %) and Medians (%)** | | | | | | |  |  |  |
|  | Range | | |  | Median | | |  |  |  |
|  | Healthy | Acute Diarrhea | Chronic Diarrhea |  | Healthy | Acute Diarrhea | Chronic Diarrhea |  | Kruskal Wallace P-value | Kruskal Wallace adjusted P-value |
| **Cellular Processes** | 1.90-5.23 | 1.93-3.92 | 1.63-3.49 |  | 2.63 | 2.58 | 2.34 |  | 0.0783 | 0.1566 |
| Cell Growth and Death | 0.46-0.61 | 0.37-0.90 | 0.32-0.60 |  | 0.51 | 0.50 | 0.52 |  | 0.2716 | 0.3783 |
| *Apoptosis* | 0.00-0.00 | 0.00-0.04 | 0.00-0.04 |  | 0.00 | 0.00 | 0.00 |  | 0.0046 | 0.0526 |
| *Cell cycle - Caulobacter* | 0.46-0.61 | 0.36-0.78 | 0.31-0.60 |  | 0.51 | 0.49 | 0.52 |  | 0.2442 | 0.3800 |
| *Meiosis - yeast* | 0.00-0.00 | 0.00-0.02 | 0.00-0.01 |  | 0.00 | 0.00 | 0.00 |  | 0.4453 | 0.5577 |
| *p53 signaling pathway* | 0.00-0.00 | 0.00-0.05 | 0.00-0.00 |  | 0.00 | 0.00 | 0.00 |  | 0.6149 | 0.6852 |
| Cell Motility | 1.09-4.55 | 1.10-3.11 | 0.83-2.92 |  | 1.77 | 1.79 | 1.56 |  | 0.2242 | 0.3363 |
| *Bacterial chemotaxis* | 0.18-1.07 | 0.23-0.62 | 0.12-0.62 |  | 0.42 | 0.36 | 0.35 |  | 0.2609 | 0.3899 |
| *Bacterial motility proteins* | 0.41-2.08 | 0.32-1.38 | 0.25-1.57 |  | 0.69 | 0.79 | 0.57 |  | 0.1861 | 0.3220 |
| *Cytoskeleton proteins* | 0.32-0.46 | 0.19-0.46 | 0.13-0.46 |  | 0.39 | 0.35 | 0.36 |  | 0.0339 | 0.1511 |
| *Flagellar assembly* | 0.15-0.94 | 0.11-0.80 | 0.08-0.81 |  | 0.32 | 0.31 | 0.24 |  | 0.0953 | 0.2161 |
| Transport and Catabolism | 0.13-0.52 | 0.15-0.47 | 0.09-0.41 |  | 0.26 | 0.25 | 0.24 |  | 0.3661 | 0.4759 |
| *Lysosome* | 0.04-0.31 | 0.00-0.26 | 0.02-0.21 |  | 0.11 | 0.09 | 0.08 |  | 0.0771 | 0.1950 |
| *Peroxisome* | 0.09-0.24 | 0.12-0.25 | 0.07-0.22 |  | 0.15 | 0.16 | 0.15 |  | 0.2506 | 0.3788 |
| **Environmental Information Processing** | 10.27-15.85 | 11.17-17.23 | 10.51-18.36 |  | 12.78 | 14.47 | 14.75 |  | 0.0063 | **0.0252** |
| Membrane Transport | 8.85-13.99 | 9.43-14.84 | 9.24-16.92 |  | 11.04 | 12.70 | 12.98 |  | 0.0044 | 0.0572 |
| *ABC transporters* | 2.23-4.31 | 2.55-4.15 | 2.38-4.16 |  | 2.98 | 3.45 | 3.49 |  | 0.0568 | 0.1867 |
| *Bacterial secretion system* | 0.51-0.72 | 0.49-1.22 | 0.44-0.93 |  | 0.57 | 0.58 | 0.54 |  | 0.0114 | 0.0810 |
| *Phosphotransferase system (PTS)* | 0.21-1.32 | 0.25-1.40 | 0.33-2.16 |  | 0.36 | 0.58 | 0.62 |  | 0.0001 | **0.0053** |
| *Secretion system* | 0.90-1.43 | 0.89-2.04 | 0.91-2.39 |  | 1.07 | 1.18 | 1.07 |  | 0.3595 | 0.4799 |
| *Transporters* | 4.31-7.72 | 4.75-7.71 | 4.64-9.43 |  | 5.91 | 6.96 | 7.16 |  | 0.0094 | 0.0824 |
| Signal Transduction | 1.10-2.09 | 1.15-2.45 | 1.06-2.78 |  | 1.48 | 1.67 | 1.41 |  | 0.0466 | 0.1515 |
| *Calcium signaling pathway* | 0.00-0.00 | 0.00-0.00 | 0.00-0.00 |  | 0.00 | 0.00 | 0.00 |  | 0.6107 | 0.6835 |
| *MAPK signaling pathway - yeast* | 0.00-0.00 | 0.00-0.00 | 0.00-0.00 |  | 0.00 | 0.00 | 0.00 |  | 0.9725 | 0.9762 |
| *Phosphatidylinositol signaling system* | 0.02-0.07 | 0.02-0.07 | 0.02-0.07 |  | 0.05 | 0.04 | 0.05 |  | 0.9009 | 0.9184 |
| *Two-component system* | 0.08-0.12 | 0.07-0.15 | 0.07-0.11 |  | 0.09 | 0.10 | 0.09 |  | 0.0536 | 0.1880 |
| Signaling Molecules and Interaction | 0.13-0.27 | 0.12-0.24 | 0.12-0.29 |  | 0.20 | 0.18 | 0.20 |  | 0.4311 | 0.5254 |
| *Bacterial toxins* | 0.98-1.99 | 1.01-2.29 | 0.93-2.66 |  | 1.30 | 1.56 | 1.29 |  | 0.1584 | 0.2976 |
| *Cellular antigens* | 0.08-0.16 | 0.07-0.20 | 0.07-0.20 |  | 0.12 | 0.12 | 0.12 |  | 0.3120 | 0.4388 |
| *Glycan bindng proteins* | 0.00-0.00 | 0.00-0.00 | 0.00-0.01 |  | 0.00 | 0.00 | 0.00 |  | 0.0577 | 0.1873 |
| *Ion channels* | 0.01-0.04 | 0.00-0.04 | 0.00-0.10 |  | 0.02 | 0.02 | 0.02 |  | 0.7766 | 0.8170 |
| **Genetic Information Processing** | 18.93-22.05 | 16.12-23.62 | 16.29-23.45 |  | 19.98 | 19.27 | 20.02 |  | 0.1291 | 0.2066 |
| Folding, Sorting and Degradation | 2.24-2.77 | 2.09-3.17 | 2.15-2.80 |  | 2.52 | 2.37 | 2.36 |  | 0.0357 | 0.1266 |
| *Chaperones and folding catalysts* | 0.89-1.22 | 0.76-1.15 | 0.82-1.26 |  | 1.04 | 0.98 | 0.95 |  | 0.0762 | 0.1946 |
| *Proteasome* | 0.04-0.05 | 0.02-0.07 | 0.00-0.06 |  | 0.05 | 0.04 | 0.04 |  | 0.0731 | 0.1962 |
| *Protein export* | 0.56-0.70 | 0.51-0.97 | 0.45-0.67 |  | 0.60 | 0.58 | 0.59 |  | 0.0451 | 0.1671 |
| *Protein processing in endoplasmic reticulum* | 0.05-0.10 | 0.02-0.09 | 0.03-0.09 |  | 0.07 | 0.06 | 0.06 |  | 0.0203 | 0.1027 |
| *RNA degradation* | 0.43-0.53 | 0.38-0.56 | 0.38-0.54 |  | 0.48 | 0.43 | 0.44 |  | 0.0019 | **0.0294** |
| *Sulfur relay system* | 0.20-0.32 | 0.20-0.37 | 0.18-0.42 |  | 0.26 | 0.29 | 0.25 |  | 0.0172 | 0.0942 |
| *Ubiquitin system* | 0.00-0.03 | 0.00-0.03 | 0.00-0.03 |  | 0.01 | 0.01 | 0.00 |  | 0.0360 | 0.1503 |
| Replication and Repair | 8.32-10.27 | 6.88-10.56 | 7.01-11.37 |  | 9.01 | 8.74 | 9.00 |  | 0.1366 | 0.2422 |
| *Base excision repair* | 0.38-0.53 | 0.36-0.52 | 0.37-0.56 |  | 0.44 | 0.44 | 0.45 |  | 0.3774 | 0.4938 |
| *Chromosome* | 1.48-1.82 | 1.33-1.72 | 1.37-1.88 |  | 1.58 | 1.54 | 1.55 |  | 0.0624 | 0.1803 |
| *DNA repair and recombination proteins* | 2.51-3.20 | 2.28-3.35 | 2.36-3.62 |  | 2.86 | 2.79 | 2.90 |  | 0.2126 | 0.3584 |
| *DNA replication* | 0.60-0.79 | 0.50-0.90 | 0.49-0.97 |  | 0.70 | 0.65 | 0.68 |  | 0.0857 | 0.2031 |
| *DNA replication proteins* | 1.12-1.47 | 0.91-1.51 | 0.93-1.60 |  | 1.25 | 1.21 | 1.24 |  | 0.0887 | 0.2064 |
| *Homologous recombination* | 0.86-1.10 | 0.64-1.15 | 0.70-1.20 |  | 0.97 | 0.93 | 0.97 |  | 0.1242 | 0.2592 |
| *Mismatch repair* | 0.81-0.98 | 0.58-1.03 | 0.58-1.10 |  | 0.86 | 0.80 | 0.84 |  | 0.0498 | 0.1770 |
| *Non-homologous end-joining* | 0.00-0.02 | 0.00-0.03 | 0.00-0.02 |  | 0.00 | 0.00 | 0.00 |  | 0.3524 | 0.4777 |
| *Nucleotide excision repair* | 0.36-0.47 | 0.26-0.45 | 0.23-0.51 |  | 0.40 | 0.37 | 0.40 |  | 0.1371 | 0.2671 |
| Transcription | 2.09-3.10 | 2.21-3.15 | 2.47-3.16 |  | 2.72 | 2.86 | 2.82 |  | 0.0201 | 0.1307 |
| *Basal transcription factors* | 0.00-0.00 | 0.00-0.01 | 0.00-0.00 |  | 0.00 | 0.00 | 0.00 |  | 0.1773 | 0.3151 |
| *RNA polymerase* | 0.12-0.19 | 0.12-0.27 | 0.12-0.27 |  | 0.17 | 0.16 | 0.17 |  | 0.0611 | 0.1869 |
| *Transcription factors* | 1.07-1.91 | 1.15-2.11 | 1.31-2.23 |  | 1.49 | 1.79 | 1.81 |  | 0.0016 | **0.0281** |
| *Transcription machinery* | 0.81-1.21 | 0.66-1.12 | 0.51-1.05 |  | 0.10 | 0.92 | 0.90 |  | 0.0261 | 0.1271 |
| Translation | 5.28-6.42 | 4.18-7.35 | 4.12-6.95 |  | 5.70 | 5.36 | 5.87 |  | 0.0545 | 0.1635 |
| *Aminoacyl-tRNA biosynthesis* | 1.03-1.41 | 0.82-1.71 | 0.73-1.53 |  | 1.22 | 1.12 | 1.23 |  | 0.0418 | 0.1617 |
| *RNA transport* | 0.10-0.17 | 0.08-0.17 | 0.09-0.21 |  | 0.14 | 0.13 | 0.14 |  | 0.4809 | 0.5910 |
| *Ribosome* | 2.12-2.79 | 1.59-3.35 | 1.53-2.94 |  | 2.35 | 2.24 | 2.45 |  | 0.1102 | 0.2376 |
| *Ribosome Biogenesis* | 1.29-1.58 | 1.17-1.53 | 1.28-1.61 |  | 1.40 | 1.35 | 1.45 |  | 0.0079 | 0.0770 |
| *Ribosome biogenesis in eukaryotes* | 0.04-0.05 | 0.04-0.06 | 0.04-0.08 |  | 0.05 | 0.05 | 0.05 |  | 0.0920 | 0.2122 |
| *Translation factors* | 0.47-0.64 | 0.35-0.64 | 0.38-0.64 |  | 0.55 | 0.52 | 0.55 |  | 0.3567 | 0.4786 |
| *mRNA surveillance pathway* | 0.00-0.00 | 0.00-0.00 | 0.00-0.00 |  | 0.00 | 0.00 | 0.00 |  | 0.1308 | 0.2606 |
| **Human Diseases** | 0.64-0.96 | 0.62-1.88 | 0.63-1.06 |  | 0.73 | 0.73 | 0.71 |  | 0.7667 | 0.7667 |
| Cancers | 0.08-0.14 | 0.08-0.35 | 0.07-0.12 |  | 0.10 | 0.10 | 0.10 |  | 0.7567 | 0.7976 |
| *Bladder cancer* | 0.00-0.00 | 0.00-0.01 | 0.00-0.02 |  | 0.00 | 0.00 | 0.00 |  | 0.0781 | 0.1956 |
| *Colorectal cancer* | 0.00-0.00 | 0.00-0.04 | 0.00-0.00 |  | 0.00 | 0.00 | 0.00 |  | 0.6069 | 0.6880 |
| *Pathways in cancer* | 0.04-0.07 | 0.04-0.16 | 0.03-0.06 |  | 0.05 | 0.05 | 0.05 |  | 0.1609 | 0.2959 |
| *Prostate cancer* | 0.04-0.06 | 0.02-0.05 | 0.00-0.05 |  | 0.05 | 0.04 | 0.04 |  | 0.0107 | 0.0853 |
| *Renal cell carcinoma* | 0.00-0.02 | 0.00-0.06 | 0.00-0.05 |  | 0.00 | 0.01 | 0.01 |  | 0.0124 | 0.0815 |
| Cardiovascular Diseases | 0.00-0.00 | 0.00-0.04 | 0.00-0.00 |  | 0.00 | 0.00 | 0.00 |  | 0.9026 | 0.9026 |
| *Hypertrophic cardiomyopathy* | 0.00-0.00 | 0.00-0.00 | 0.00-0.00 |  | 0.00 | 0.00 | 0.00 |  | 0.6153 | 0.6828 |
| *Viral myocarditis* | 0.00-0.00 | 0.00-0.04 | 0.00-0.00 |  | 0.00 | 0.00 | 0.00 |  | 0.6729 | 0.7223 |
| Immune System Diseases | 0.03-0.07 | 0.02-0.07 | 0.03-0.11 |  | 0.05 | 0.05 | 0.05 |  | 0.4047 | 0.5091 |
| *Primary immunodeficiency* | 0.03-0.07 | 0.01-0.07 | 0.03-0.11 |  | 0.05 | 0.05 | 0.05 |  | 0.3636 | 0.4805 |
| *Systemic lupus erythematosus* | 0.00-0.00 | 0.00-0.00 | 0.00-0.00 |  | 0.00 | 0.00 | 0.00 |  | 0.6153 | 0.6799 |
| Infectious Diseases | 0.34-0.42 | 0.32-0.61 | 0.33-0.64 |  | 0.37 | 0.39 | 0.37 |  | 0.5321 | 0.6104 |
| *African trypanosomiasis* | 0.00-0.00 | 0.00-0.05 | 0.00-0.03 |  | 0.00 | 0.00 | 0.00 |  | 0.1640 | 0.2975 |
| *Amoebiasis* | 0.00-0.02 | 0.00-0.02 | 0.00-0.02 |  | 0.01 | 0.01 | 0.01 |  | 0.4500 | 0.5609 |
| *Bacterial invasion of epithelial cells* | 0.00-0.00 | 0.00-0.02 | 0.00-0.03 |  | 0.00 | 0.00 | 0.00 |  | 0.2134 | 0.3552 |
| *Chagas disease (American trypanosomiasis)* | 0.00-0.00 | 0.00-0.04 | 0.00-0.03 |  | 0.00 | 0.00 | 0.00 |  | 0.0236 | 0.1171 |
| *Epithelial cell signaling in Helicobacter pylori infection* | 0.09-0.14 | 0.03-0.15 | 0.01-0.12 |  | 0.1^a^ | 0.08^b^ | 0.09^b^ |  | 0.0004 | **0.0175** |
| *Influenza A* | 0.00-0.00 | 0.00-0.04 | 0.00-0.00 |  | 0.00 | 0.00 | 0.00 |  | 0.8137 | 0.8492 |
| *Pathogenic Escherichia coli infection* | 0.00-0.00 | 0.00-0.02 | 0.00-0.01 |  | 0.00 | 0.00 | 0.00 |  | 0.0123 | 0.0829 |
| *Pertussis* | 0.00-0.07 | 0.00-0.19 | 0.00-0.25 |  | 0.01 | 0.02 | 0.01 |  | 0.1363 | 0.2675 |
| *Shigellosis* | 0.00-0.00 | 0.00-0.01 | 0.00-0.00 |  | 0.00 | 0.00 | 0.00 |  | 0.0134 | 0.0839 |
| *Staphylococcus aureus infection* | 0.00-0.01 | 0.00-0.11 | 0.00-0.21 |  | 0.00^a^ | 0.01^b^ | 0.01^ab^ |  | 0.0001 | **0.0263** |
| *Toxoplasmosis* | 0.00-0.00 | 0.00-0.04 | 0.00-0.00 |  | 0.00 | 0.00 | 0.00 |  | 0.6070 | 0.6822 |
| *Tuberculosis* | 0.13-0.18 | 0.11-0.23 | 0.09-0.17 |  | 0.15 | 0.14 | 0.15 |  | 0.0196 | 0.1011 |
| *Vibrio cholerae infection* | 0.00-0.00 | 0.00-0.00 | 0.00-0.00 |  | 0.00 | 0.00 | 0.00 |  | 0.2757 | 0.4028 |
| *Vibrio cholerae pathogenic cycle* | 0.06-0.11 | 0.05-0.13 | 0.06-0.14 |  | 0.09 | 0.09 | 0.08 |  | 0.2492 | 0.3788 |
| Metabolic Diseases | 0.09-0.12 | 0.08-0.12 | 0.08-0.13 |  | 0.10 | 0.10 | 0.10 |  | 0.0829 | 0.2021 |
| *Type I diabetes mellitus* | 0.05-0.06 | 0.04-0.07 | 0.04-0.06 |  | 0.05 | 0.05 | 0.05 |  | 0.5886 | 0.6760 |
| *Type II diabetes mellitus* | 0.04-0.06 | 0.03-0.06 | 0.04-0.07 |  | 0.05 | 0.05 | 0.05 |  | 0.0090 | 0.0845 |
| Neurodegenerative Diseases | 0.06-0.28 | 0.06-0.78 | 0.07-0.17 |  | 0.10 | 0.09 | 0.09 |  | 0.4736 | 0.5597 |
| *Alzheimer's disease* | 0.04-0.13 | 0.04-0.24 | 0.03-0.06 |  | 0.05 | 0.05 | 0.05 |  | 0.0111 | 0.0834 |
| *Amyotrophic lateral sclerosis (ALS)* | 0.00-0.02 | 0.00-0.12 | 0.00-0.05 |  | 0.01 | 0.01 | 0.01 |  | 0.2465 | 0.3791 |
| *Huntington's disease* | 0.01-0.08 | 0.01-0.24 | 0.01-0.06 |  | 0.03 | 0.03 | 0.02 |  | 0.2279 | 0.3655 |
| *Parkinson's disease* | 0.00-0.05 | 0.00-0.16 | 0.00-0.01 |  | 0.00 | 0.00 | 0.00 |  | 0.0005 | **0.0146** |
| *Prion diseases* | 0.00-0.01 | 0.00-0.02 | 0.00-0.02 |  | 0.00 | 0.01 | 0.01 |  | 0.0009 | **0.0237** |
| **Metabolism** | 45.20-51.28 | 44.35-51.15 | 43.18-49.34 |  | 48.37 | 47.68 | 47.07 |  | 0.0635 | 0.1693 |
| Amino Acid Metabolism | 9.14-10.63 | 8.17-10.19 | 6.44-10.38 |  | 9.74 | 9.40 | 9.44 |  | 0.1252 | 0.2441 |
| *Alanine, aspartate and glutamate metabolism* | 0.89-1.24 | 0.66-1.26 | 0.83-1.18 |  | 1.11 | 1.07 | 1.07 |  | 0.1197 | 0.2539 |
| *Amino acid related enzymes* | 1.43-1.69 | 1.27-1.75 | 1.14-1.66 |  | 1.53 | 1.45 | 1.51 |  | 0.1215 | 0.2556 |
| *Arginine and proline metabolism* | 1.02-1.32 | 0.86-1.31 | 0.67-1.32 |  | 1.24 | 1.17 | 1.15 |  | 0.0413 | 0.1646 |
| *Cysteine and methionine metabolism* | 0.86-1.05 | 0.70-1.03 | 0.77-1.10 |  | 0.94 | 0.91 | 0.96 |  | 0.0675 | 0.1889 |
| *Glycine, serine and threonine metabolism* | 0.78-0.91 | 0.71-0.94 | 0.48-0.89 |  | 0.84 | 0.83 | 0.82 |  | 0.5858 | 0.6787 |
| *Histidine metabolism* | 0.54-0.82 | 0.44-0.75 | 0.17-0.78 |  | 0.66 | 0.60 | 0.61 |  | 0.0616 | 0.1862 |
| *Lysine biosynthesis* | 0.77-0.96 | 0.61-0.95 | 0.55-0.93 |  | 0.84 | 0.80 | 0.85 |  | 0.1272 | 0.2614 |
| *Lysine degradation* | 0.08-0.15 | 0.09-0.31 | 0.06-0.30 |  | 0.11 | 0.14 | 0.11 |  | 0.0012 | **0.0243** |
| *Phenylalanine metabolism* | 0.15-0.23 | 0.15-0.26 | 0.07-0.26 |  | 0.18 | 0.20 | 0.17 |  | 0.0443 | 0.1689 |
| *Phenylalanine, tyrosine and tryptophan biosynthesis* | 0.76-1.06 | 0.42-0.96 | 0.13-1.00 |  | 0.88 | 0.80 | 0.83 |  | 0.0193 | 0.1015 |
| *Tryptophan metabolism* | 0.09-0.16 | 0.09-0.39 | 0.09-0.31 |  | 0.11 | 0.14 | 0.13 |  | 0.0026 | **0.0342** |
| *Tyrosine metabolism* | 0.26-0.37 | 0.31-0.59 | 0.31-0.47 |  | 0.33 | 0.35 | 0.34 |  | 0.0154 | 0.0880 |
| *Valine, leucine and isoleucine biosynthesis* | 0.55-0.86 | 0.53-0.84 | 0.33-0.84 |  | 0.75 | 0.71 | 0.69 |  | 0.5406 | 0.6347 |
| *Valine, leucine and isoleucine degradation* | 0.14-0.26 | 0.16-0.60 | 0.16-0.33 |  | 0.21 | 0.23 | 0.20 |  | 0.0339 | 0.1486 |
| Biosynthesis of Other Secondary Metabolites | 0.81-1.23 | 0.57-1.11 | 0.57-1.02 |  | 0.96 | 0.89 | 0.90 |  | 0.0049 | **0.0478** |
| *Betalain biosynthesis* | 0.00-0.00 | 0.00-0.00 | 0.00-0.00 |  | 0.00 | 0.00 | 0.00 |  | 0.2195 | 0.3631 |
| *Butirosin and neomycin biosynthesis* | 0.05-0.11 | 0.03-0.09 | 0.01-0.08 |  | 0.07 | 0.06 | 0.06 |  | 0.0832 | 0.2007 |
| *Caffeine metabolism* | 0.00-0.00 | 0.00-0.01 | 0.00-0.00 |  | 0.00 | 0.00 | 0.00 |  | 0.0009 | **0.0215** |
| *Flavone and flavonol biosynthesis* | 0.00-0.03 | 0.00-0.01 | 0.00-0.01 |  | 0.00 | 0.00 | 0.00 |  | 0.2748 | 0.4060 |
| *Flavonoid biosynthesis* | 0.00-0.02 | 0.00-0.02 | 0.00-0.02 |  | 0.00 | 0.00 | 0.00 |  | 0.9263 | 0.9406 |
| *Indole alkaloid biosynthesis* | 0.00-0.00 | 0.00-0.00 | 0.00-0.00 |  | 0.00 | 0.00 | 0.00 |  | 0.5107 | 0.6161 |
| *Isoflavonoid biosynthesis* | 0.00-0.00 | 0.00-0.01 | 0.00-0.00 |  | 0.00 | 0.00 | 0.00 |  | 0.0832 | 0.1989 |
| *Isoquinoline alkaloid biosynthesis* | 0.03-0.10 | 0.04-0.08 | 0.00-0.09 |  | 0.06 | 0.06 | 0.06 |  | 0.2748 | 0.4038 |
| *Novobiocin biosynthesis* | 0.11-0.20 | 0.10-0.17 | 0.02-0.19 |  | 0.14 | 0.14 | 0.14 |  | 0.5626 | 0.6576 |
| *Penicillin and cephalosporin biosynthesis* | 0.01-0.05 | 0.00-0.05 | 0.00-0.06 |  | 0.02 | 0.03 | 0.02 |  | 0.8407 | 0.8671 |
| *Phenylpropanoid biosynthesis* | 0.10-0.36 | 0.05-0.19 | 0.06-0.19 |  | 0.14 | 0.15 | 0.14 |  | 0.2572 | 0.3865 |
| *Stilbenoid, diarylheptanoid and gingerol biosynthesis* | 0.00-0.02 | 0.00-0.02 | 0.00-0.01 |  | 0.00 | 0.00 | 0.00 |  | 0.3016 | 0.4311 |
| *Streptomycin biosynthesis* | 0.27-0.39 | 0.14-0.41 | 0.17-0.38 |  | 0.34 | 0.31 | 0.31 |  | 0.3405 | 0.4713 |
| *Tropane, piperidine and pyridine alkaloid biosynthesis* | 0.10-0.18 | 0.10-0.15 | 0.01-0.16 |  | 0.12 | 0.12 | 0.12 |  | 0.0111 | 0.0811 |
| *beta-Lactam resistance* | 0.01-0.05 | 0.01-0.05 | 0.00-0.05 |  | 0.03 | 0.03 | 0.03 |  | 0.5935 | 0.6757 |
| Carbohydrate Metabolism | 9.82-11.77 | 8.37-11.71 | 9.69-12.49 |  | 10.83 | 10.79 | 10.67 |  | 0.6782 | 0.7557 |
| *Amino sugar and nucleotide sugar metabolism* | 1.40-1.99 | 0.83-1.72 | 1.05-2.10 |  | 1.57 | 1.50 | 1.55 |  | 0.1274 | 0.2577 |
| *Ascorbate and aldarate metabolism* | 0.07-0.13 | 0.08-0.24 | 0.06-0.21 |  | 0.10 | 0.13 | 0.12 |  | 0.0092 | 0.0834 |
| *Butanoate metabolism* | 0.54-0.85 | 0.49-0.83 | 0.49-0.93 |  | 0.60 | 0.70 | 0.63 |  | 0.0156 | 0.0873 |
| *C5-Branched dibasic acid metabolism* | 0.21-0.41 | 0.22-0.36 | 0.05-0.38 |  | 0.32 | 0.30 | 0.29 |  | 0.1814 | 0.3181 |
| *Citrate cycle (TCA cycle)* | 0.48-0.87 | 0.50-1.16 | 0.34-0.81 |  | 0.67 | 0.65 | 0.53 |  | 0.0822 | 0.2002 |
| *Fructose and mannose metabolism* | 0.76-1.31 | 0.50-1.28 | 0.72-1.88 |  | 1.07 | 1.05 | 1.02 |  | 0.8143 | 0.8465 |
| *Galactose metabolism* | 0.57-1.01 | 0.29-0.91 | 0.59-1.05 |  | 0.78 | 0.78 | 0.78 |  | 0.6277 | 0.6822 |
| *Glycolysis / Gluconeogenesis* | 0.97-1.26 | 0.99-1.26 | 0.94-1.70 |  | 1.10 | 1.09 | 1.11 |  | 0.5403 | 0.6372 |
| *Glyoxylate and dicarboxylate metabolism* | 0.43-0.70 | 0.45-0.68 | 0.24-0.71 |  | 0.55 | 0.56 | 0.52 |  | 0.0712 | 0.1951 |
| *Inositol phosphate metabolism* | 0.07-0.14 | 0.06-0.20 | 0.06-0.15 |  | 0.10 | 0.12 | 0.09 |  | 0.0714 | 0.1936 |
| *Pentose and glucuronate interconversions* | 0.38-0.72 | 0.21-0.68 | 0.32-0.64 |  | 0.54 | 0.57 | 0.51 |  | 0.1243 | 0.2574 |
| *Pentose phosphate pathway* | 0.76-1.01 | 0.44-1.05 | 0.69-1.20 |  | 0.89 | 0.92 | 0.88 |  | 0.9471 | 0.9580 |
| *Propanoate metabolism* | 0.44-0.53 | 0.44-0.75 | 0.41-0.62 |  | 0.49 | 0.54 | 0.50 |  | 0.0590 | 0.1870 |
| *Pyruvate metabolism* | 0.88-1.14 | 0.91-1.18 | 0.81-1.18 |  | 1.00 | 1.06 | 1.03 |  | 0.0616 | 0.1841 |
| *Starch and sucrose metabolism* | 0.78-1.43 | 0.43-1.18 | 0.66-1.26 |  | 1.04 | 0.98 | 1.02 |  | 0.1350 | 0.2670 |
| Energy Metabolism | 5.54-6.31 | 4.94-6.90 | 4.84-6.29 |  | 6.03 | 5.76 | 5.82 |  | 0.0297 | 0.1655 |
| *Carbon fixation in photosynthetic organisms* | 0.61-0.74 | 0.48-0.74 | 0.46-0.77 |  | 0.67 | 0.64 | 0.66 |  | 0.2270 | 0.3663 |
| *Carbon fixation pathways in prokaryotes* | 0.83-1.29 | 0.88-1.30 | 0.77-1.21 |  | 1.09 | 1.02 | 0.98 |  | 0.0617 | 0.1823 |
| *Methane metabolism* | 1.13-1.49 | 0.63-1.50 | 0.87-1.49 |  | 1.29 | 1.28 | 1.22 |  | 0.4895 | 0.5933 |
| *Nitrogen metabolism* | 0.65-0.82 | 0.54-0.87 | 0.47-0.92 |  | 0.72 | 0.73 | 0.68 |  | 0.1007 | 0.2244 |
| *Oxidative phosphorylation* | 0.99-1.32 | 1.02-2.15 | 0.77-1.32 |  | 1.18 | 1.07 | 1.08 |  | 0.0543 | 0.1879 |
| *Photosynthesis* | 0.24-0.55 | 0.25-0.73 | 0.22-0.48 |  | 0.42 | 0.38 | 0.43 |  | 0.0462 | 0.1688 |
| *Photosynthesis - antenna proteins* | 0.00-0.00 | 0.00-0.07 | 0.00-0.00 |  | 0.00 | 0.00 | 0.00 |  | 0.3678 | 0.4837 |
| *Photosynthesis proteins* | 0.25-0.56 | 0.28-0.81 | 0.24-0.48 |  | 0.42 | 0.38 | 0.43 |  | 0.0565 | 0.1881 |
| *Sulfur metabolism* | 0.22-0.35 | 0.21-0.32 | 0.18-0.31 |  | 0.27 | 0.27 | 0.26 |  | 0.4629 | 0.5716 |
| Enzyme Families | 2.06-2.46 | 2.01-2.39 | 2.06-2.53 |  | 2.14 | 2.16 | 2.22 |  | 0.0637 | 0.1775 |
| *Peptidases* | 1.77-2.26 | 1.59-2.14 | 1.53-2.33 |  | 1.88 | 1.85 | 1.94 |  | 0.0588 | 0.1886 |
| *Protein kinases* | 0.20-0.35 | 0.23-0.51 | 0.19-0.52 |  | 0.28 | 0.31 | 0.28 |  | 0.0150 | 0.0877 |
| Glycan Biosynthesis and Metabolism | 1.65-3.52 | 1.42-3.25 | 1.46-3.29 |  | 2.68 | 2.37 | 2.08 |  | 0.0882 | 0.1911 |
| *Glycosaminoglycan biosynthesis - chondroitin sulfate* | 0.03-0.23 | 0.00-0.19 | 0.01-0.16 |  | 0.09 | 0.06 | 0.05 |  | 0.0753 | 0.1942 |
| *Glycosaminoglycan degradation* | 0.02-0.17 | 0.00-0.14 | 0.00-0.13 |  | 0.07 | 0.04 | 0.04 |  | 0.0555 | 0.1871 |
| *Glycosphingolipid biosynthesis - ganglio series* | 0.05-0.24 | 0.00-0.20 | 0.04-0.18 |  | 0.13 | 0.10 | 0.09 |  | 0.0372 | 0.1529 |
| *Glycosphingolipid biosynthesis - globo series* | 0.00-0.00 | 0.00-0.00 | 0.00-0.00 |  | 0.00 | 0.00 | 0.00 |  | 0.0009 | **0.0197** |
| *Glycosyltransferases* | 0.26-0.41 | 0.24-0.49 | 0.25-0.62 |  | 0.33 | 0.33 | 0.33 |  | 0.6902 | 0.7379 |
| *Lipopolysaccharide biosynthesis* | 0.07-0.69 | 0.04-0.58 | 0.01-0.55 |  | 0.31 | 0.25 | 0.19 |  | 0.1420 | 0.2706 |
| *Lipopolysaccharide biosynthesis proteins* | 0.14-0.85 | 0.09-0.82 | 0.08-0.84 |  | 0.43 | 0.37 | 0.30 |  | 0.2208 | 0.3629 |
| *N-Glycan biosynthesis* | 0.01-0.03 | 0.00-0.03 | 0.00-0.03 |  | 0.02 | 0.02 | 0.01 |  | 0.2314 | 0.3666 |
| *Other glycan degradation* | 0.13-0.66 | 0.02-0.61 | 0.07-0.52 |  | 0.30 | 0.29 | 0.24 |  | 0.1381 | 0.2671 |
| *Peptidoglycan biosynthesis* | 0.74-0.99 | 0.65-1.02 | 0.64-0.99 |  | 0.80 | 0.79 | 0.87 |  | 0.1172 | 0.2506 |
| *Various types of N-glycan biosynthesis* | 0.00-0.00 | 0.00-0.00 | 0.00-0.00 |  | 0.00 | 0.00 | 0.00 |  | 0.2128 | 0.3565 |
| Lipid Metabolism | 2.31-3.01 | 2.45-3.26 | 2.21-2.87 |  | 2.66 | 2.80 | 2.70 |  | 0.0333 | 0.1443 |
| *Arachidonic acid metabolism* | 0.01-0.08 | 0.01-0.06 | 0.01-0.08 |  | 0.02 | 0.03 | 0.03 |  | 0.0071 | 0.0718 |
| *Biosynthesis of unsaturated fatty acids* | 0.09-0.12 | 0.09-0.22 | 0.08-0.25 |  | 0.10 | 0.12 | 0.11 |  | 0.5928 | 0.6779 |
| *Ether lipid metabolism* | 0.00-0.00 | 0.00-0.01 | 0.00-0.01 |  | 0.00 | 0.00 | 0.00 |  | 0.0028 | **0.0351** |
| *Fatty acid biosynthesis* | 0.32-0.51 | 0.32-0.65 | 0.34-0.50 |  | 0.45 | 0.46 | 0.45 |  | 0.1526 | 0.2887 |
| *Fatty acid metabolism* | 0.12-0.23 | 0.17-0.48 | 0.17-0.45 |  | 0.18^a^ | 0.24^b^ | 0.22^ab^ |  | 0.2478 | 0.3789 |
| *Glycerolipid metabolism* | 0.29-0.47 | 0.31-0.47 | 0.29-0.53 |  | 0.36 | 0.38 | 0.40 |  | 0.0001 | **0.0132** |
| *Glycerophospholipid metabolism* | 0.47-0.62 | 0.49-0.67 | 0.48-0.63 |  | 0.53 | 0.55 | 0.54 |  | 0.3329 | 0.4657 |
| *Linoleic acid metabolism* | 0.05-0.14 | 0.04-0.10 | 0.02-0.10 |  | 0.07 | 0.07 | 0.07 |  | 0.4206 | 0.5396 |
| *Lipid biosynthesis proteins* | 0.39-0.61 | 0.43-0.75 | 0.34-0.60 |  | 0.54 | 0.56 | 0.53 |  | 0.4281 | 0.5439 |
| *Primary bile acid biosynthesis* | 0.02-0.06 | 0.01-0.04 | 0.01-0.05 |  | 0.04 | 0.03 | 0.03 |  | 0.0328 | 0.1487 |
| *Secondary bile acid biosynthesis* | 0.02-0.06 | 0.01-0.04 | 0.01-0.05 |  | 0.04 | 0.03 | 0.03 |  | 0.3509 | 0.4782 |
| *Sphingolipid metabolism* | 0.09-0.46 | 0.02-0.36 | 0.05-0.31 |  | 0.21 | 0.19 | 0.19 |  | 0.3079 | 0.4354 |
| *Steroid biosynthesis* | 0.00-0.00 | 0.00-0.02 | 0.00-0.00 |  | 0.00 | 0.00 | 0.00 |  | 0.1416 | 0.2718 |
| *Steroid hormone biosynthesis* | 0.00-0.07 | 0.00-0.04 | 0.00-0.04 |  | 0.02 | 0.02 | 0.01 |  | 0.2251 | 0.3654 |
| *Synthesis and degradation of ketone bodies* | 0.02-0.09 | 0.02-0.11 | 0.02-0.11 |  | 0.03 | 0.04 | 0.04 |  | 0.0448 | 0.1683 |
| *alpha-Linolenic acid metabolism* | 0.00-0.02 | 0.00-0.04 | 0.00-0.06 |  | 0.00 | 0.01 | 0.00 |  | 0.7325 | 0.7768 |
| Metabolism of Cofactors and Vitamins | 3.86-4.78 | 3.72-4.75 | 2.62-4.83 |  | 4.46 | 4.34 | 4.25 |  | 0.0862 | 0.1978 |
| *Biotin metabolism* | 0.13-0.26 | 0.11-0.23 | 0.01-0.20 |  | 0.19 | 0.15 | 0.14 |  | 0.0024 | **0.0332** |
| *Folate biosynthesis* | 0.26-0.55 | 0.31-0.56 | 0.24-0.51 |  | 0.42 | 0.41 | 0.38 |  | 0.2878 | 0.4159 |
| *Lipoic acid metabolism* | 0.01-0.07 | 0.01-0.13 | 0.01-0.06 |  | 0.02 | 0.04 | 0.02 |  | 0.0110 | 0.0851 |
| *Nicotinate and nicotinamide metabolism* | 0.41-0.50 | 0.25-0.50 | 0.35-0.53 |  | 0.44 | 0.43 | 0.44 |  | 0.2850 | 0.4141 |
| *One carbon pool by folate* | 0.53-0.79 | 0.42-0.73 | 0.41-0.83 |  | 0.63 | 0.57 | 0.63 |  | 0.0999 | 0.2246 |
| *Pantothenate and CoA biosynthesis* | 0.53-0.75 | 0.44-0.70 | 0.45-0.69 |  | 0.64 | 0.61 | 0.61 |  | 0.0619 | 0.1809 |
| *Porphyrin and chlorophyll metabolism* | 0.54-1.19 | 0.59-1.25 | 0.06-1.33 |  | 0.91 | 1.01 | 0.90 |  | 0.2051 | 0.3480 |
| *Retinol metabolism* | 0.01-0.05 | 0.01-0.09 | 0.01-0.06 |  | 0.02 | 0.03 | 0.02 |  | 0.3805 | 0.4954 |
| *Riboflavin metabolism* | 0.15-0.31 | 0.19-0.33 | 0.16-0.34 |  | 0.26 | 0.25 | 0.24 |  | 0.5868 | 0.6769 |
| *Thiamine metabolism* | 0.48-0.59 | 0.36-0.57 | 0.37-0.60 |  | 0.52 | 0.49 | 0.52 |  | 0.0640 | 0.1810 |
| *Ubiquinone and other terpenoid-quinone biosynthesis* | 0.08-0.30 | 0.07-0.58 | 0.02-0.44 |  | 0.17 | 0.20 | 0.14 |  | 0.2393 | 0.3746 |
| *Vitamin B6 metabolism* | 0.17-0.24 | 0.11-0.24 | 0.12-0.25 |  | 0.20 | 0.18 | 0.19 |  | 0.0547 | 0.1868 |
| Metabolism of Other Amino Acids | 1.22-1.67 | 1.32-1.61 | 1.33-1.73 |  | 1.54 | 1.52 | 1.51 |  | 0.6782 | 0.7347 |
| *Cyanoamino acid metabolism* | 0.24-0.51 | 0.16-0.34 | 0.18-0.34 |  | 0.30 | 0.28 | 0.28 |  | 0.0341 | 0.1470 |
| *D-Alanine metabolism* | 0.08-0.13 | 0.09-0.15 | 0.08-0.21 |  | 0.10 | 0.10 | 0.11 |  | 0.0282 | 0.1324 |
| *D-Arginine and D-ornithine metabolism* | 0.00-0.02 | 0.00-0.02 | 0.00-0.01 |  | 0.00 | 0.00 | 0.00 |  | 0.8626 | 0.8862 |
| *D-Glutamine and D-glutamate metabolism* | 0.13-0.19 | 0.11-0.18 | 0.11-0.19 |  | 0.15 | 0.15 | 0.15 |  | 0.7801 | 0.8174 |
| *Glutathione metabolism* | 0.10-0.27 | 0.14-0.34 | 0.14-0.38 |  | 0.18 | 0.22 | 0.21 |  | 0.1860 | 0.3240 |
| *Phosphonate and phosphinate metabolism* | 0.02-0.07 | 0.03-0.08 | 0.01-0.09 |  | 0.05 | 0.05 | 0.05 |  | 0.5252 | 0.6279 |
| *Selenocompound metabolism* | 0.34-0.43 | 0.32-0.41 | 0.30-0.44 |  | 0.38 | 0.38 | 0.38 |  | 0.4425 | 0.5568 |
| *Taurine and hypotaurine metabolism* | 0.09-0.16 | 0.08-0.17 | 0.07-0.20 |  | 0.11 | 0.11 | 0.11 |  | 0.5673 | 0.6602 |
| *beta-Alanine metabolism* | 0.11-0.22 | 0.15-0.31 | 0.02-0.31 |  | 0.19 | 0.21 | 0.18 |  | 0.0750 | 0.1992 |
| Metabolism of Terpenoids and Polyketides | 1.41-1.87 | 1.48-1.79 | 1.39-1.86 |  | 1.64 | 1.59 | 1.60 |  | 0.3175 | 0.4270 |
| *Biosynthesis of 12-, 14- and 16-membered macrolides* | 0.00-0.00 | 0.00-0.00 | 0.00-0.00 |  | 0.00 | 0.00 | 0.00 |  | 0.4809 | 0.5883 |
| *Biosynthesis of ansamycins* | 0.09-0.20 | 0.03-0.16 | 0.03-0.16 |  | 0.12 | 0.12 | 0.11 |  | 0.3367 | 0.4685 |
| *Biosynthesis of siderophore group nonribosomal peptides* | 0.00-0.04 | 0.00-0.13 | 0.00-0.12 |  | 0.01 | 0.03 | 0.02 |  | 0.0267 | 0.1277 |
| *Biosynthesis of type II polyketide products* | 0.00-0.00 | 0.00-0.00 | 0.00-0.00 |  | 0.00 | 0.00 | 0.00 |  | 0.2938 | 0.4222 |
| *Biosynthesis of vancomycin group antibiotics* | 0.05-0.09 | 0.02-0.08 | 0.04-0.08 |  | 0.07 | 0.06 | 0.06 |  | 0.0283 | 0.1306 |
| *Carotenoid biosynthesis* | 0.00-0.01 | 0.00-0.04 | 0.00-0.02 |  | 0.00 | 0.00 | 0.00 |  | 0.4244 | 0.5418 |
| *Geraniol degradation* | 0.01-0.07 | 0.01-0.19 | 0.00-0.19 |  | 0.03 | 0.04 | 0.03 |  | 0.0596 | 0.1866 |
| *Limonene and pinene degradation* | 0.04-0.10 | 0.05-0.33 | 0.04-0.16 |  | 0.07 | 0.08 | 0.08 |  | 0.2361 | 0.3718 |
| *Polyketide sugar unit biosynthesis* | 0.17-0.26 | 0.04-0.28 | 0.10-0.26 |  | 0.22 | 0.20 | 0.20 |  | 0.1289 | 0.2588 |
| *Prenyltransferases* | 0.26-0.41 | 0.26-0.46 | 0.23-0.43 |  | 0.32 | 0.29 | 0.31 |  | 0.2663 | 0.3957 |
| *Terpenoid backbone biosynthesis* | 0.51-0.71 | 0.42-0.67 | 0.35-0.69 |  | 0.57 | 0.56 | 0.60 |  | 0.0752 | 0.1978 |
| *Tetracycline biosynthesis* | 0.07-0.18 | 0.09-0.20 | 0.06-0.20 |  | 0.13 | 0.15 | 0.13 |  | 0.1651 | 0.2974 |
| *Zeatin biosynthesis* | 0.05-0.08 | 0.03-0.07 | 0.03-0.08 |  | 0.06 | 0.05 | 0.05 |  | 0.0485 | 0.1747 |
| Nucleotide Metabolism | 3.73-4.72 | 3.29-4.61 | 3.38-5.34 |  | 4.08 | 4.04 | 4.10 |  | 0.1443 | 0.2447 |
| *Purine metabolism* | 2.05-2.53 | 1.88-2.52 | 2.06-3.04 |  | 2.25 | 2.20 | 2.24 |  | 0.1273 | 0.2595 |
| *Pyrimidine metabolism* | 1.66-2.19 | 1.42-2.10 | 1.33-2.31 |  | 1.84 | 1.83 | 1.86 |  | 0.2281 | 0.3636 |
| Xenobiotics Biodegradation and Metabolism | 1.06-1.86 | 1.45-2.90 | 1.36-2.22 |  | 1.55^a^ | 1.70^b^ | 1.67^b^ |  | 0.0023 | **0.0449** |
| *1,1,1-Trichloro-2,2-bis(4-chlorophenyl)ethane (DDT) degradation* | 0.00-0.00 | 0.00-0.00 | 0.00-0.01 |  | 0.00 | 0.00 | 0.00 |  | 0.0952 | 0.2177 |
| *Aminobenzoate degradation* | 0.07-0.13 | 0.08-0.34 | 0.08-0.16 |  | 0.11 | 0.11 | 0.11 |  | 0.6353 | 0.6876 |
| *Atrazine degradation* | 0.00-0.06 | 0.00-0.07 | 0.00-0.03 |  | 0.02 | 0.01 | 0.01 |  | 0.0752 | 0.1958 |
| *Benzoate degradation* | 0.13-0.32 | 0.16-0.46 | 0.16-0.40 |  | 0.19 | 0.22 | 0.23 |  | 0.0064 | 0.0701 |
| *Bisphenol degradation* | 0.06-0.15 | 0.05-0.11 | 0.02-0.12 |  | 0.09 | 0.09 | 0.08 |  | 0.5259 | 0.6258 |
| *Caprolactam degradation* | 0.00-0.02 | 0.00-0.10 | 0.00-0.15 |  | 0.01 | 0.02 | 0.01 |  | 0.0012 | **0.0225** |
| *Chloroalkane and chloroalkene degradation* | 0.11-0.27 | 0.13-0.26 | 0.10-0.27 |  | 0.16 | 0.19 | 0.18 |  | 0.0631 | 0.1804 |
| *Chlorocyclohexane and chlorobenzene degradation* | 0.00-0.03 | 0.00-0.07 | 0.00-0.04 |  | 0.01 | 0.01 | 0.01 |  | 0.1017 | 0.2248 |
| *Dioxin degradation* | 0.03-0.07 | 0.02-0.13 | 0.03-0.16 |  | 0.04^a^ | 0.06^ab^ | 0.07^b^ |  | 0.0001 | **0.0088** |
| *Drug metabolism - cytochrome P450* | 0.01-0.04 | 0.01-0.12 | 0.01-0.10 |  | 0.02 | 0.03 | 0.02 |  | 0.0104 | 0.0882 |
| *Drug metabolism - other enzymes* | 0.25-0.41 | 0.14-0.37 | 0.21-0.40 |  | 0.32 | 0.33 | 0.33 |  | 0.6379 | 0.6876 |
| *Ethylbenzene degradation* | 0.01-0.07 | 0.02-0.18 | 0.02-0.07 |  | 0.05 | 0.04 | 0.04 |  | 0.4350 | 0.5500 |
| *Fluorobenzoate degradation* | 0.00-0.00 | 0.00-0.02 | 0.00-0.03 |  | 0.00^a^ | 0.00^b^ | 0.00^ab^ |  | 0.0004 | **0.0150** |
| *Metabolism of xenobiotics by cytochrome P450* | 0.01-0.04 | 0.01-0.12 | 0.00-0.10 |  | 0.02 | 0.03 | 0.02 |  | 0.0135 | 0.0826 |
| *Naphthalene degradation* | 0.05-0.17 | 0.11-0.38 | 0.10-0.22 |  | 0.13 | 0.14 | 0.14 |  | 0.3565 | 0.4808 |
| *Nitrotoluene degradation* | 0.05-0.20 | 0.01-0.15 | 0.00-0.15 |  | 0.09 | 0.10 | 0.09 |  | 0.5174 | 0.6214 |
| *Polycyclic aromatic hydrocarbon degradation* | 0.09-0.16 | 0.05-0.18 | 0.03-0.16 |  | 0.11 | 0.11 | 0.11 |  | 0.3892 | 0.5018 |
| *Styrene degradation* | 0.01-0.05 | 0.01-0.05 | 0.01-0.05 |  | 0.01 | 0.02 | 0.02 |  | 0.0067 | 0.0705 |
| *Toluene degradation* | 0.04-0.13 | 0.04-0.25 | 0.04-0.38 |  | 0.08 | 0.09 | 0.07 |  | 0.3031 | 0.4309 |
| *Xylene degradation* | 0.01-0.07 | 0.01-0.10 | 0.03-0.14 |  | 0.03^a^ | 0.06^ab^ | 0.07^b^ |  | 0.0001 | **0.0066** |
| **None** | 0.15-0.23 | 0.09-0.22 | 0.07-0.24 |  | 0.19 | 0.18 | 0.18 |  | 0.6348 | 0.7255 |
| **Organismal Systems** | 0.64-0.84 | 0.52-0.86 | 0.33-0.81 |  | 0.76 | 0.70 | 0.65 |  | 0.0062 | **0.0496** |
| Circulatory System | 0.00-0.05 | 0.00-0.12 | 0.00-0.00 |  | 0.00 | 0.00 | 0.00 |  | 0.0005 | **0.0195** |
| *Cardiac muscle contraction* | 0.00-0.05 | 0.00-0.12 | 0.00-0.00 |  | 0.00 | 0.00 | 0.00 |  | 0.0019 | **0.0278** |
| Digestive System | 0.02-0.11 | 0.01-0.09 | 0.00-0.12 |  | 0.05 | 0.04 | 0.04 |  | 0.1194 | 0.2451 |
| *Carbohydrate digestion and absorption* | 0.01-0.03 | 0.00-0.03 | 0.00-0.03 |  | 0.02 | 0.01 | 0.01 |  | 0.1689 | 0.3022 |
| *Mineral absorption* | 0.00-0.03 | 0.00-0.03 | 0.00-0.03 |  | 0.00 | 0.01 | 0.00 |  | 0.8290 | 0.8584 |
| *Protein digestion and absorption* | 0.01-0.06 | 0.00-0.05 | 0.00-0.06 |  | 0.03 | 0.02 | 0.02 |  | 0.1067 | 0.2319 |
| Endocrine System | 0.22-0.35 | 0.15-0.37 | 0.12-0.33 |  | 0.29 | 0.28 | 0.27 |  | 0.2180 | 0.3401 |
| *Adipocytokine signaling pathway* | 0.03-0.10 | 0.01-0.10 | 0.00-0.11 |  | 0.07 | 0.06 | 0.06 |  | 0.6188 | 0.6809 |
| *Insulin signaling pathway* | 0.06-0.13 | 0.04-0.12 | 0.06-0.09 |  | 0.08 | 0.08 | 0.07 |  | 0.1605 | 0.2973 |
| *PPAR signaling pathway* | 0.07-0.13 | 0.06-0.14 | 0.06-0.14 |  | 0.10 | 0.10 | 0.10 |  | 0.6192 | 0.6785 |
| *Progesterone-mediated oocyte maturation* | 0.04-0.05 | 0.02-0.05 | 0.00-0.05 |  | 0.05 | 0.04 | 0.04 |  | 0.0387 | 0.1566 |
| *Renin-angiotensin system* | 0.00-0.00 | 0.00-0.01 | 0.00-0.00 |  | 0.00 | 0.00 | 0.00 |  | 0.2249 | 0.3674 |
| Environmental Adaptation | 0.14-0.23 | 0.12-0.21 | 0.09-0.21 |  | 0.17 | 0.15 | 0.16 |  | 0.0298 | 0.1453 |
| *Circadian rhythm - plant* | 0.00-0.00 | 0.00-0.01 | 0.00-0.00 |  | 0.00 | 0.00 | 0.00 |  | 0.3480 | 0.4792 |
| *Plant-pathogen interaction* | 0.14-0.22 | 0.11-0.20 | 0.10-0.22 |  | 0.17 | 0.15 | 0.16 |  | 0.0349 | 0.1480 |
| Excretory System | 0.01-0.04 | 0.00-0.04 | 0.00-0.04 |  | 0.02 | 0.02 | 0.01 |  | 0.0182 | 0.1420 |
| *Proximal tubule bicarbonate reclamation* | 0.01-0.04 | 0.01-0.04 | 0.00-0.04 |  | 0.02 | 0.02 | 0.01 |  | 0.0139 | 0.0831 |
| *Vasopressin-regulated water reabsorption* | 0.00-0.00 | 0.00-0.00 | 0.00-0.00 |  | 0.00 | 0.00 | 0.00 |  | 0.4809 | 0.5855 |
| Immune System | 0.07-0.11 | 0.05-0.10 | 0.00-0.11 |  | 0.10 | 0.09 | 0.09 |  | 0.0337 | 0.1314 |
| *Antigen processing and presentation* | 0.03-0.05 | 0.02-0.05 | 0.00-0.05 |  | 0.04 | 0.04 | 0.04 |  | 0.0116 | 0.0803 |
| *NOD-like receptor signaling pathway* | 0.04-0.06 | 0.03-0.06 | 0.00-0.05 |  | 0.05 | 0.04 | 0.04 |  | 0.0787 | 0.1953 |
| *RIG-I-like receptor signaling pathway* | 0.00-0.00 | 0.00-0.01 | 0.00-0.01 |  | 0.00 | 0.00 | 0.00 |  | 0.0016 | **0.0263** |
| Nervous System | 0.08-0.13 | 0.07-0.14 | 0.06-0.13 |  | 0.11 | 0.11 | 0.11 |  | 0.2487 | 0.3592 |
| *Glutamatergic synapse* | 0.09-0.13 | 0.07-0.14 | 0.06-0.13 |  | 0.11 | 0.11 | 0.11 |  | 0.1611 | 0.2942 |
| **Unclassified** | 13.32-14.98 | 12.77-16.56 | 12.85-17.52 |  | 14.03 | 14.37 | 14.13 |  | 0.5052 | 0.6736 |
| Cellular Processes and Signaling | 3.69-4.54 | 3.67-5.29 | 2.87-5.38 |  | 4.17 | 4.23 | 4.10 |  | 0.1296 | 0.2407 |
| *Cell division* | 0.06-0.11 | 0.05-0.11 | 0.01-0.11 |  | 0.09 | 0.08 | 0.08 |  | 0.7732 | 0.8167 |
| *Cell motility and secretion* | 0.11-0.30 | 0.12-0.28 | 0.10-0.25 |  | 0.18 | 0.17 | 0.16 |  | 0.3498 | 0.4792 |
| *Electron transfer carriers* | 0.00-0.04 | 0.00-0.10 | 0.00-0.15 |  | 0.02 | 0.03 | 0.02 |  | 0.0413 | 0.1621 |
| *Germination* | 0.01-0.06 | 0.00-0.20 | 0.00-0.10 |  | 0.03 | 0.04 | 0.03 |  | 0.7167 | 0.7631 |
| *Inorganic ion transport and metabolism* | 0.13-0.23 | 0.14-0.38 | 0.09-0.48 |  | 0.16 | 0.19 | 0.17 |  | 0.0104 | 0.0855 |
| *Membrane and intracellular structural molecules* | 0.36-0.83 | 0.32-0.94 | 0.24-0.95 |  | 0.59 | 0.60 | 0.48 |  | 0.1811 | 0.3197 |
| *Other ion-coupled transporters* | 1.13-1.59 | 1.12-1.82 | 1.03-1.86 |  | 1.31 | 1.41 | 1.33 |  | 0.3605 | 0.4788 |
| *Other transporters* | 0.25-0.31 | 0.19-0.30 | 0.14-0.32 |  | 0.27 | 0.28 | 0.27 |  | 0.9519 | 0.9592 |
| *Pores ion channels* | 0.22-0.54 | 0.16-0.65 | 0.12-0.63 |  | 0.38 | 0.35 | 0.29 |  | 0.1906 | 0.3276 |
| *Signal transduction mechanisms* | 0.33-0.57 | 0.35-0.57 | 0.38-0.67 |  | 0.44 | 0.49 | 0.50 |  | 0.1031 | 0.2260 |
| *Sporulation* | 0.29-1.11 | 0.11-1.12 | 0.01-1.15 |  | 0.60 | 0.62 | 0.62 |  | 0.9992 | 0.9992 |
| Genetic Information Processing | 2.42-2.96 | 2.27-3.15 | 2.45-3.20 |  | 2.61 | 2.64 | 2.70 |  | 0.1498 | 0.2434 |
| *Protein folding and associated processing* | 0.47-0.82 | 0.53-0.84 | 0.35-0.79 |  | 0.64 | 0.62 | 0.62 |  | 0.8688 | 0.8891 |
| *Replication, recombination and repair proteins* | 0.69-0.91 | 0.61-1.22 | 0.69-1.28 |  | 0.77 | 0.80 | 0.82 |  | 0.0682 | 0.1888 |
| *Restriction enzyme* | 0.17-0.27 | 0.03-0.25 | 0.06-0.34 |  | 0.23 | 0.17 | 0.18 |  | 0.0004 | **0.0132** |
| *Transcription related proteins* | 0.00-0.01 | 0.00-0.04 | 0.00-0.05 |  | 0.00 | 0.00 | 0.00 |  | 0.0037 | **0.0442** |
| *Translation proteins* | 0.84-1.03 | 0.78-1.02 | 0.78-1.05 |  | 0.92 | 0.89 | 0.93 |  | 0.0885 | 0.2078 |
| Metabolism | 2.10-2.73 | 1.84-3.15 | 2.13-3.18 |  | 2.39 | 2.56 | 2.46 |  | 0.0780 | 0.2028 |
| *Amino acid metabolism* | 0.08-0.28 | 0.12-0.36 | 0.13-0.30 |  | 0.20 | 0.23 | 0.22 |  | 0.0809 | 0.1988 |
| *Biosynthesis and biodegradation of secondary metabolites* | 0.02-0.07 | 0.02-0.13 | 0.01-0.15 |  | 0.05 | 0.06 | 0.05 |  | 0.0131 | 0.0840 |
| *Carbohydrate metabolism* | 0.11-0.22 | 0.10-0.21 | 0.10-0.21 |  | 0.18 | 0.18 | 0.17 |  | 0.6201 | 0.6767 |
| *Energy metabolism* | 0.74-1.01 | 0.55-1.01 | 0.51-1.01 |  | 0.90 | 0.90 | 0.87 |  | 0.5343 | 0.6330 |
| *Glycan biosynthesis and metabolism* | 0.02-0.07 | 0.01-0.11 | 0.00-0.15 |  | 0.03 | 0.04 | 0.03 |  | 0.2443 | 0.3779 |
| *Lipid metabolism* | 0.10-0.15 | 0.11-0.17 | 0.07-0.17 |  | 0.12 | 0.13 | 0.13 |  | 0.2019 | 0.3448 |
| *Metabolism of cofactors and vitamins* | 0.09-0.20 | 0.08-0.23 | 0.08-0.23 |  | 0.11 | 0.13 | 0.12 |  | 0.4545 | 0.5638 |
| *Nucleotide metabolism* | 0.02-0.08 | 0.02-0.14 | 0.02-0.16 |  | 0.03 | 0.05 | 0.05 |  | 0.0601 | 0.1860 |
| *Others* | 0.69-1.04 | 0.72-1.40 | 0.76-1.38 |  | 0.90 | 0.94 | 0.97 |  | 0.0190 | 0.1020 |
| Poorly Characterized | 4.60-5.36 | 4.59-5.57 | 4.58-5.75 |  | 4.92 | 5.01 | 4.97 |  | 0.7570 | 0.7769 |
| *Function unknown* | 1.09-1.40 | 1.10-1.93 | 1.04-2.23 |  | 1.22 | 1.29 | 1.24 |  | 0.1593 | 0.2971 |
| *General function prediction only* | 3.35-4.23 | 3.23-3.85 | 3.34-3.93 |  | 3.69 | 3.66 | 3.73 |  | 0.3809 | 0.4935 |
| *Medians not sharing a common superscript are significantly different (p<0.05 based on Dunn's multiple comparisons test).* | | | | | | | | | | |
